# Supplementary material for: CDK phosphorylation of Sfr1 downregulates Rad51 function in late-meiotic homolog invasions
Source: EMBO J. 2024 Aug 22;43(19):4356–83. doi: 10.1038/s44318-024-00205-2 (PMC11445502; doi:10.1038/s44318-024-00205-2)
Supplement: Supplementary file 1 — Appendix [file 44318_2024_205_MOESM1_ESM.pdf]

Appendix

CDK phosphorylation of Sfr1 downregulates Rad51 function in late-meiotic homolog invasions

Inés Palacios-Blanco, Lucía Gómez, María Bort, Nina Mayerova,
Silvia Bágeľová Poláková, and Cristina Martín-Castellanos.

Appendix Figure S1. Recombination proficiency of the *EGFP-sfr1* allele.

Appendix Figure S1 Legend.

Appendix Figure S2. Meiotic progression in *sfr1-7A*, *sfr1-7D* and *sfr1-WI* mutants.

Appendix Figure S2 Legend.

Appendix Figure S3. Rad51 expression in *sfr1-7A* and *sfr1-7D* phospho-mutants, and *sfr1-WI* mutant.

Appendix Figure S3 Legend.

Appendix Figure S4. *In vivo* characterization of the *sfr1-WI* allele.

Appendix Figure S4 Legend.

Appendix Figure S5. Sequence alignment of Sfr1 proteins in *Schizosaccharomyces* genus.

Appendix Figure S5 Legend.

Appendix Figure S6. Sequence alignment of fission yeast and mouse Sfr1 proteins.

Appendix Figure S6 Legend.

Appendix Figure S7. Anti-GFP and anti-Rad51 specificity.

Appendix Figure S7 Legend.

Appendix Table S1. *S. pombe* strains.

Appendix Table S1 Legend.

Appendix References.

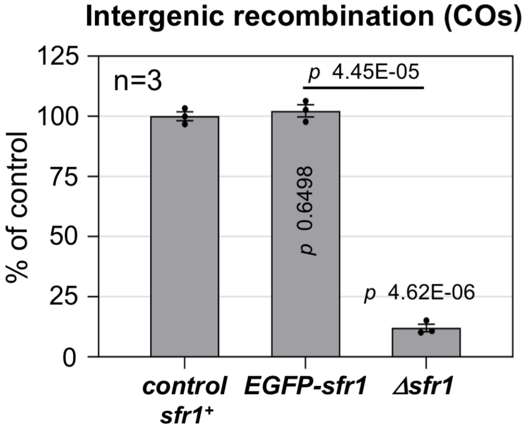

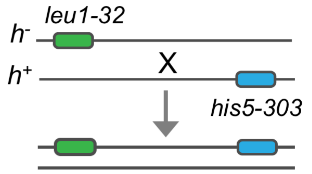

Genotype	Intergenic recombination (h^- *leu1-32* x h^+ *his5-303*) **% Leu$^+$ His$^+$** 32ºC
control sfr1$^+$ (CMC1474 x CMC737)	9.76 ± 0.18 (100) n=3
EGFP-sfr1 (CMC1630 x CMC1629)	9.99 ± 0.43 (102) n=3
Δsfr1 (CMC1427 x CMC1408)	1.18 ± 0.18 (12) n=3

Appendix Figure S1

Appendix Figure S1. Recombination proficiency of the *EGFP-sfr1* allele.

Scheme of the recombination assay is shown. Crosses of h^- *leu1-32* x h^+ *his5-303* strains were performed in SPA at 32ºC, and plated for recombination analysis twice. To control for temperature effects, crosses were done at the temperature used for synchronizing meiotic entry. Intergenic recombination (CO) levels are expressed as the percentage of Leu^+ His^+ recombinants per haploid spore colonies based on the cumulative numbers in each cross; 19-241 haploid Leu^+ His^+ recombinant colonies scored in each independent cross, and 73-637 total haploid Leu^+ His^+ recombinant colonies scored per genotype. Table shows the mean +/- SEM of 3 independent crosses, with numbers in parentheses showing the percentages relative to wild-type control. Strains used in the crosses are indicated. Graph shows the mean of the percentage relative to the control cross +/- SEM. *p* values were calculated based on Student´s t-test (unpaired, two tails). Related to Figure 1.

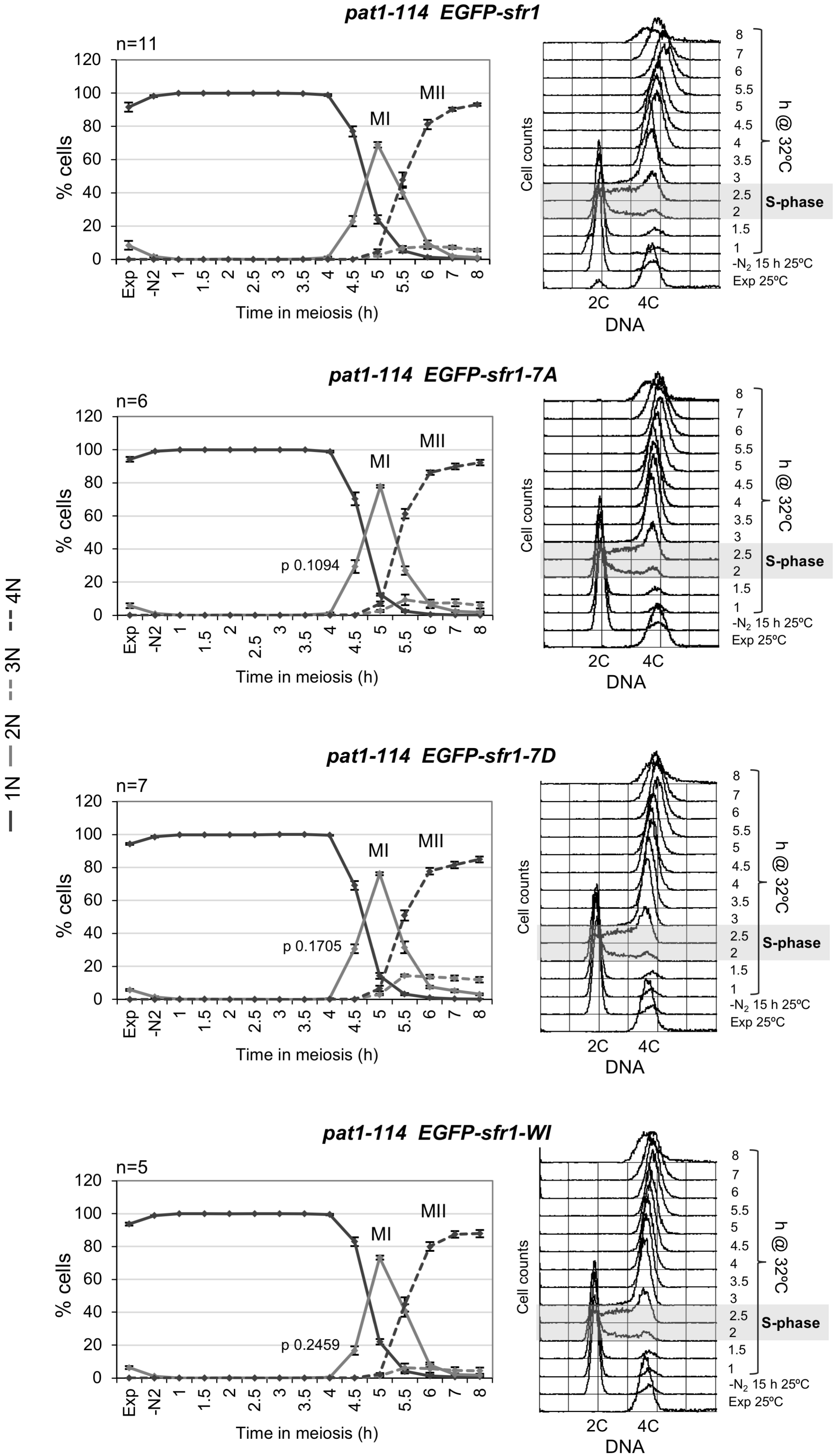

Appendix Figure S2

Appendix Figure S2. Meiotic progression in *sfr1-7A*, *sfr1-7D* and *sfr1-WI* mutants.

pat1-114 EGFP-sfr1 (CMC1649), *EGFP-sfr1-7A* (CMC1733), *EGFP-sfr1-7D* (CMC1756) and *EGFP-sfr1-WI* (CMC1769) diploid cells were induced to enter meiosis and collected at the indicated time points. Meiotic progression measured as the number of nuclei per cell is shown on the left. Data are the mean +/- S.E.M. of n independent kinetics. All the strains showed the peak of MI at 5 h after meiotic induction. *p* values at 4.5 h (meiosis I entry) were calculated based on Student´s t-test (unpaired, two tails). Each mutant was analyzed only with the *EGFP-sfr1* control in the same kinetics. FACS analysis of a representative kinetics is shown on the right; timing of DNA synthesis is highlighted. In all the kinetics the strains showed the same timing of DNA replication. Related to Figures 1, 2, 4, EV2, EV3, EV4A, Appendix S3C and Appendix S4C.

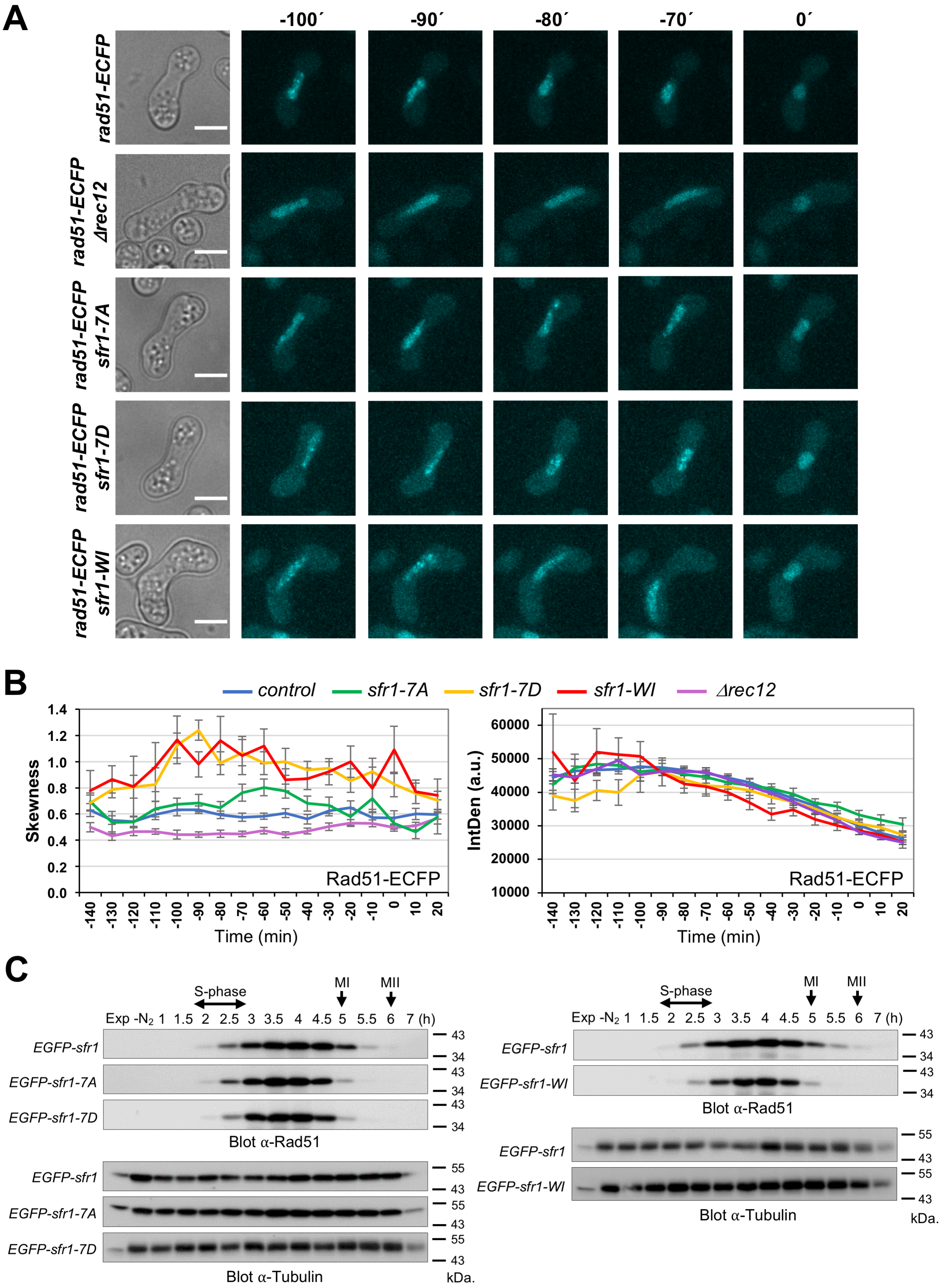

Appendix Figure S3

Appendix Figure S3. Rad51 expression in *sfr1-7A* and *sfr1-7D* phospho-mutants, and *sfr1-WI* mutant.

A Time lapse microscopy of zygotes expressing a tagged Rad51-ECFP version. Zygotes were obtained in crosses of h^{+} *rad51-ECFP-ura4*$^{+}$*-rad51* (*sfr1*$^{+}$ or mutant allele) X h^{-} (or *sfr1* mutant allele) strains: control *rad51-ECFP* (CMC1735 X CMC1826), *rad51-ECFP Δrec12* (CMC1894 X CMC1896), *rad51-ECFP sfr1-7A* (CMC1802 X CMC1794), *rad51-ECFP sfr1-7D* (CMC1738 X CMC1716) and *rad51-ECFP sfr1-WI* (CMC1776 X CMC1765). Time point 0 was set as the frame when nucleus stopped the characteristic *horsetail* movement at the end of meiotic prophase. Representative images at different time points during prophase are shown (maximum Z projections). Scale bars correspond to 5 μm. Complete time lapses are shown in Movie EV5-EV9. **B** Quantification of the skewness (left) and total intensity (right) of Rad51-ECFP in *Δrec12*, *sfr1-7A*, *sfr1-7D* and *sfr1-WI* mutants. Data are the mean +/- SEM of n independent zygotes based on the cumulative numbers of several time lapse experiments. 3-50 zygotes analyzed at each time point, and at least 9 zygotes analyzed from time point -100 min to +20 min; n and *p* values are presented in Table EV3 and EV4. **C** *pat1-114 EGFP-sfr1* (CMC1649), *EGFP-sfr1-7A* (CMC1733), *EGFP-sfr1-7D* (CMC1756) and *EGFP-sfr1-WI* (CMC1769) diploid cells were induced to enter meiosis and collected at the indicated time points. Western blot detection of Rad51 protein is shown (upper blots). The same membranes shown in Figure EV4A and Appendix Figure S4C were reused for this second western blot; therefore, tubulin detection as loading control is the same for this new WB and same blots are presented here (lower blots).

A

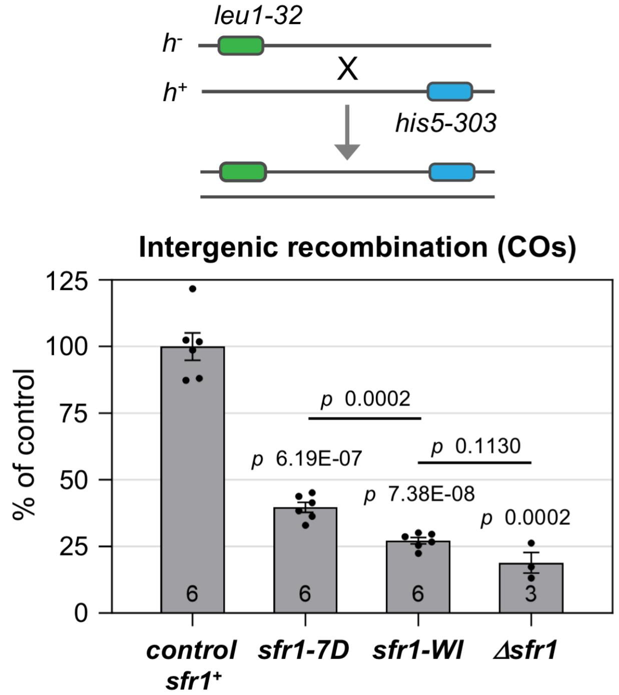

Genotype	Intergenic recombination (h^- *leu1-32* x h^+ *his5-303*) **% Leu$^+$ His$^+$** 25ºC
control sfr1$^+$ (CMC319 x CMC737)	11.15 ± 0.57 (100) n=6
sfr1-7D (CMC1380 x CMC1381)	4.39 ± 0.19 (39) n=6
sfr1-WI (CMC1767 x CMC1766)	3.01 ± 0.12 (27) n=6
control sfr1$^+$ (CMC319 x CMC737)	10.33 ± 0.55 (100) n=3
sfr1-7D (CMC1380 x CMC1381)	4.06 ± 0.17 (39) n=3
sfr1-WI (CMC1767 x CMC1766)	2.78 ± 0.09 (27) n=3
Δsfr1 (CMC1427 x CMC1408)	1.92 ± 0.33 (19) n=3

B

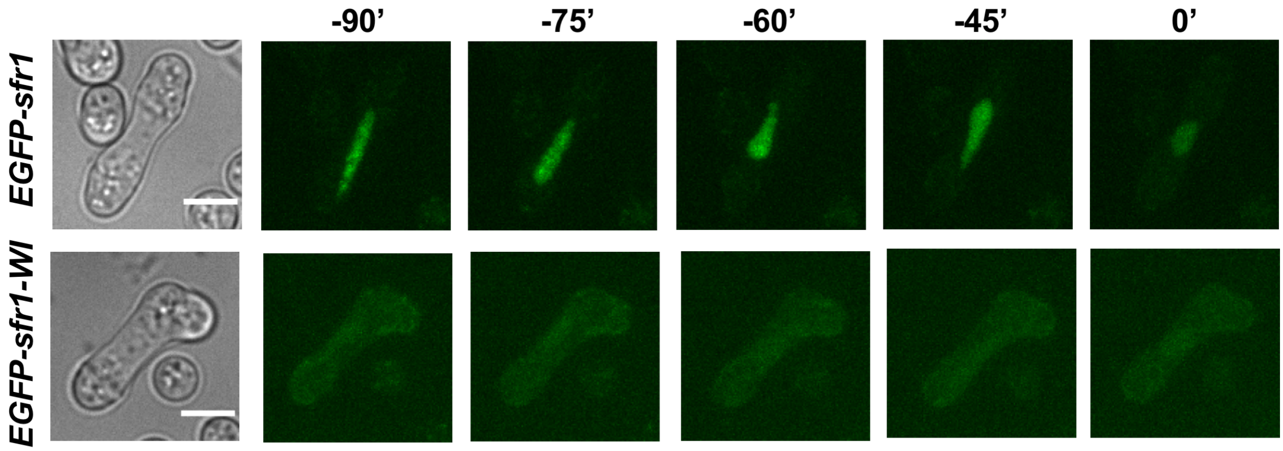

C

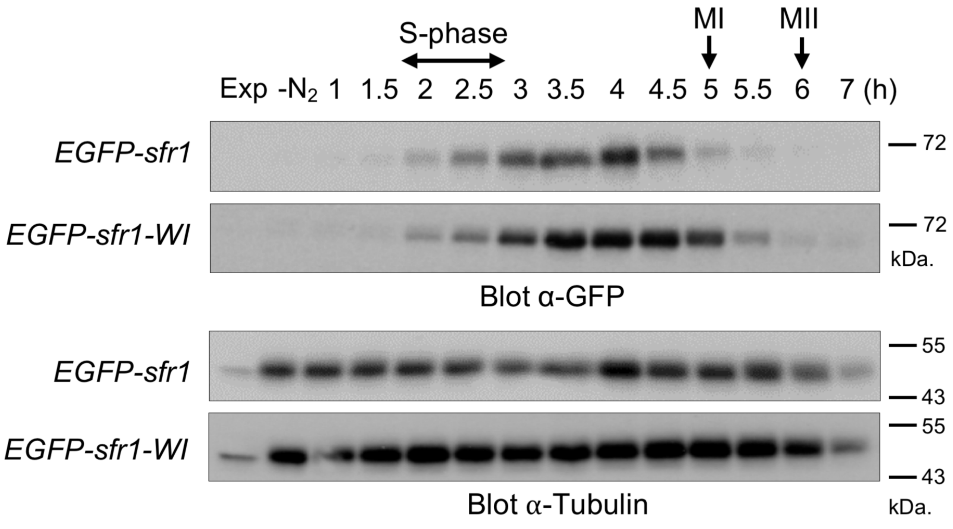

Appendix Figure S4

Appendix Figure S4. *In vivo* characterization of the *sfr1-WI* allele.

A Recombination proficiency. Scheme of the recombination assay is shown. Crosses of h^- *leu1-32* x h^+ *his5-303* strains were performed in MEA at 25ºC, and plated for recombination analysis twice. Table shows intergenic recombination (CO) levels expressed as the mean of the percentage of Leu^+ His^+ recombinants per haploid spore colonies +/- SEM of n independent crosses based on the cumulative numbers in each cross; 29-293 haploid Leu^+ His^+ recombinant colonies scored in each independent cross, and 132-1356 total haploid Leu^+ His^+ recombinant colonies scored per genotype. The numbers in parentheses are percentages relative to wild-type control. Each mutant was analyzed only with crosses in the same experiment. Strains used in the crosses are indicated. Graph shows recombination expressed as the mean of the percentage relative to the control cross +/- SEM of n independent crosses. *p* values were calculated based on Student´s t-test (unpaired, two tails). **B** *In vivo* EGFP-Sfr1-WI localization. Time lapse microscopy of zygotes expressing tagged EGFP-Sfr1 and EGFP-Sfr1-WI versions in heterozygosis. Zygotes were obtained in crosses of h^- *EGFP-sfr1* (or *EGFP-sfr1-WI* allele) X h^+ (or *sfr1-WI* allele) strains: control *EGFP-sfr1* (CMC1788 X CMC2) and *EGFP-sfr1-WI* (CMC1743 X CMC1789). Time point 0 was set as the frame when *horsetail* movement ends. 8 *EGFP-sfr1-WI* zygotes were analyzed. Representative images at different time points during prophase are shown (maximum Z projections). Scale bars correspond to 5 µm. **C** EGFP-Sfr1-WI expression. *pat1-114 EGFP-sfr1* (CMC1649) and *EGFP-sfr1-WI* (CMC1769) diploid cells were induced to enter meiosis and collected at the indicated time points. Western blot detection of EGFP-Sfr1 and EGFP-Sfr1-WI proteins is shown (upper blots); tubulin detection was used as loading control (lower blots).

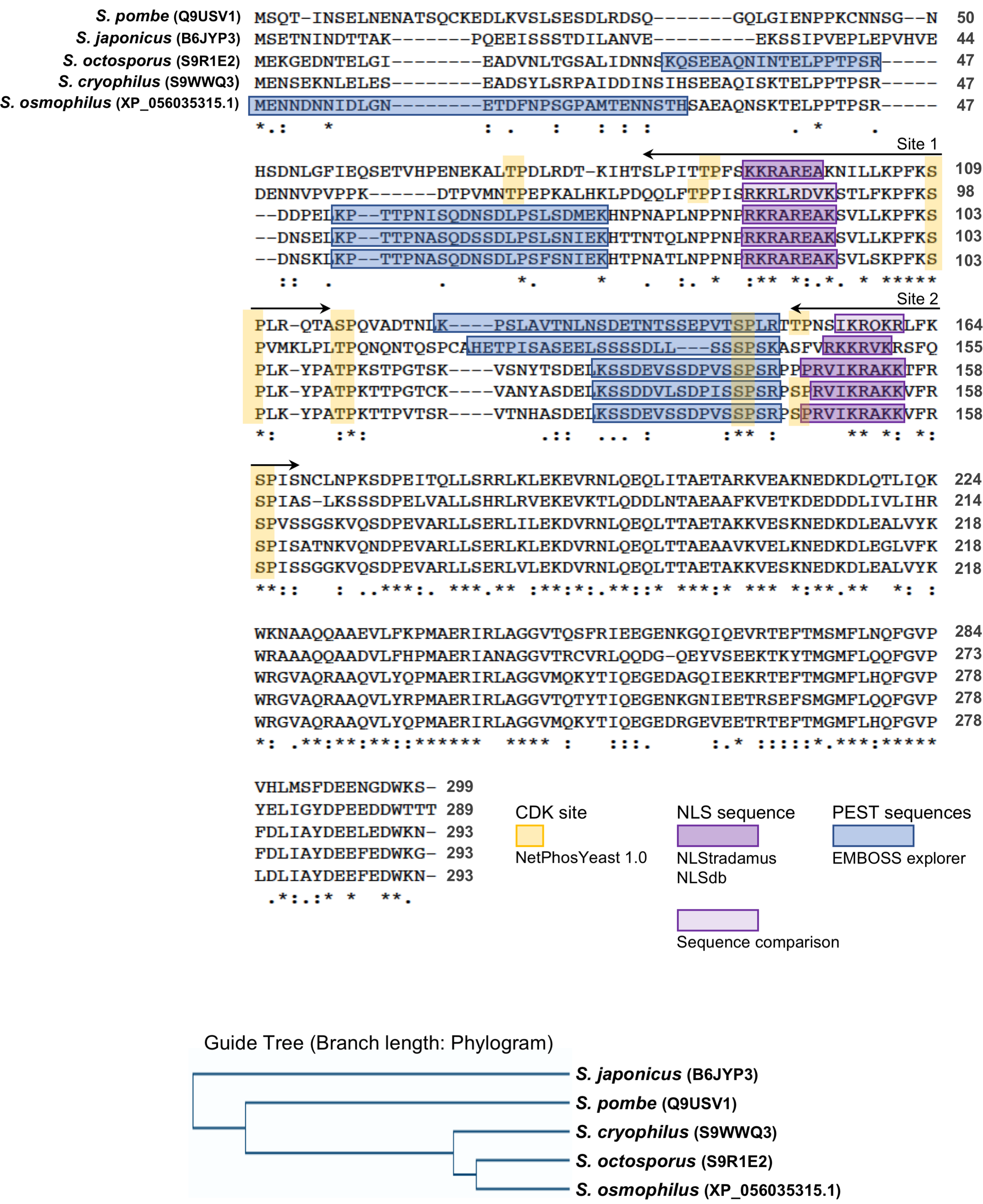

Appendix Figure S5

Appendix Figure S5. Sequence alignment of Sfr1 proteins in *Schizosaccharomyces* genus.

Sequence alignment was generated with Clustal Omega using Sfr1 homologs from *S. pombe* (UniProtKB Q9USV1), *S. japonicus* (UniProtKB B6JYP3), *S. octosporus* (UniProtKB S9R1E2), *S. cryophilus* (UniProtKB S9WWQ3) and *S. osmophilus* (XP_056035315.1). Position of Site 1 and Site 2 involved in Rad51 interaction are depicted on top of the *S. pombe* protein sequence (Argunhan *et al*, 2020). NLS signatures are highlighted in purple and were identified using NLSdb (Bernhofer *et al*, 2018) and NLStradamus (http://www.moseslab.csb.utoronto.ca/NLStradamus/) (Nguyen Ba *et al*, 2009) servers. PEST signatures are highlighted in blue and were identified using epestfind in EMBOSS explorer server (https://emboss.bioinformatics.nl/cgi-bin/emboss/epestfind). CDK sites are highlighted in orange and were predicted with high score (≥ 0.787) using NetPhosYeast 1.0 server (https://services.healthtech.dtu.dk/services/NetPhosYeast-1.0/). The generated guide tree reflecting the phylogenetic distances of the species is shown below.

```
S. pombe (Q9USV1)    MSQTINSELNENATSQCKEDLKVSLSESDLRDSQGQLGIENPPKCNNS---GNHSDNLGF 57
M. musculus (Q8BP27) MAEEGNQEF----TSK----MENS-SDSASTSPDAPQPSENPPSPPTSPAAPQTSENPPS 51
                     *::  *.*:    **:    :: * *:*    . :.     ****.   .*     : *:*

                     IEQSETVHPENEKALTPDLRDTKIHTSLPITTPFSKKRAREA-----KNILLKPFKSPLR 112
                     --------P----PTSPAVPQTRENPPSPPTSPAAPQPRENPPSPPTSPAAPQPRENPPS 99
                             *       :* : :*: :    * *:* : :   .:         .    :* :.*

                     ---QTASPQVAD---------TNLKP-------------------SLAVTNLNSDET- 138
                     PPTSPAAPQPRENPPSPHSNSSGKQPLSGTPKERLKKARSSSHSFCSVVKRMKVENDENN 159
                       . *:** :         :. :*                           :   :::.**.

                     NTSSEPVTSPLRT----TPNSIKRQK----RLFKSPISNCLNPKSDPEIT---------Q 181
                     ETLSEPGESSKEENCSKAQESLKNKDSEPGEKSSEEKNTCESKSSDTGSSNALPKESENA 219
                     :* *** * .      : :*:*.:.     .   ..   ..* . .**    :

                     LL-----SRRLKLEKEVRNLQEQLITAETARKVEAKNEDKDLQTLIQKWKNAAQQAEVL 236
                     IIREKLKQEKIRLIRQVEEKEDLLRRLKLVKMYRIKNDVTELENLIKKWRKCGQRLLCEL 279
                     ::      ..:::* ::*.: :: *    : .:   . **: .:*:.**:**::..*:     *

                     FKPMAERIRLAGGVTQSFRIEEGENKGQIQEVRTEFTMSMFLNQFGVPVHLMSFDEENGD 296
                     QSIMSED-----------------------EDEKLTLTELIDFYGIDDNLLHYNRSEEE 315
                      . *:*                        ::*:: ::: :*:  :*: ::..: :

                     WKS- 299
                     FTGV 319
                     :..
```

Proline-rich repeats | CDK site (NetPhos 3.1) | NLS sequence (NLStradamus, NLSdb) | PEST sequences (EMBOSS explorer)

Proline-rich repeats

	1	2	3	4	5
N-terminal	Ser23 0.969	Ser39 0.923	Ser55 0.797	Ser71 0.923	Ser87 0.923
C-terminal (PPSP context)	Ser35 0.933	Ser51 0.933	Ser67 0.968	Ser83 0.968	Ser99 0.968

NetPhos 3.1 score

Appendix Figure S6

Appendix Figure S6. Sequence alignment of fission yeast and mouse Sfr1 proteins.

Sequence alignment was generated with Clustal Omega using Sfr1 homologs from *S. pombe* (UniProtKB Q9USV1) and *M. musculus* (UniProtKB Q8BP27). Proline-rich repeats are highlighted in green in the mouse protein (Akamatsu & Jasin, 2010). NLS signatures are highlighted in purple and were identified using NLSdb (Bernhofer *et al*, 2018) and NLStradamus (http://www.moseslab.csb.utoronto.ca/NLStradamus/) (Nguyen Ba *et al*, 2009) servers. PEST signatures are highlighted in blue and were identified using epestfind in EMBOSS explorer server (https://emboss.bioinformatics.nl/cgi-bin/emboss/epestfind). CDK sites are highlighted in orange and were predicted with high score using NetPhos 3.1 server (https://services.healthtech.dtu.dk/services/NetPhos-3.1/). In the table below, the scores of the conserved CDK sites at the N-terminal part of each of the 5 Proline-repeats are indicated. At the C-terminal part of the repeats, a CDK site in the PPSP context is also predicted with a high score (these sites are not highlighted in the sequence alignment).

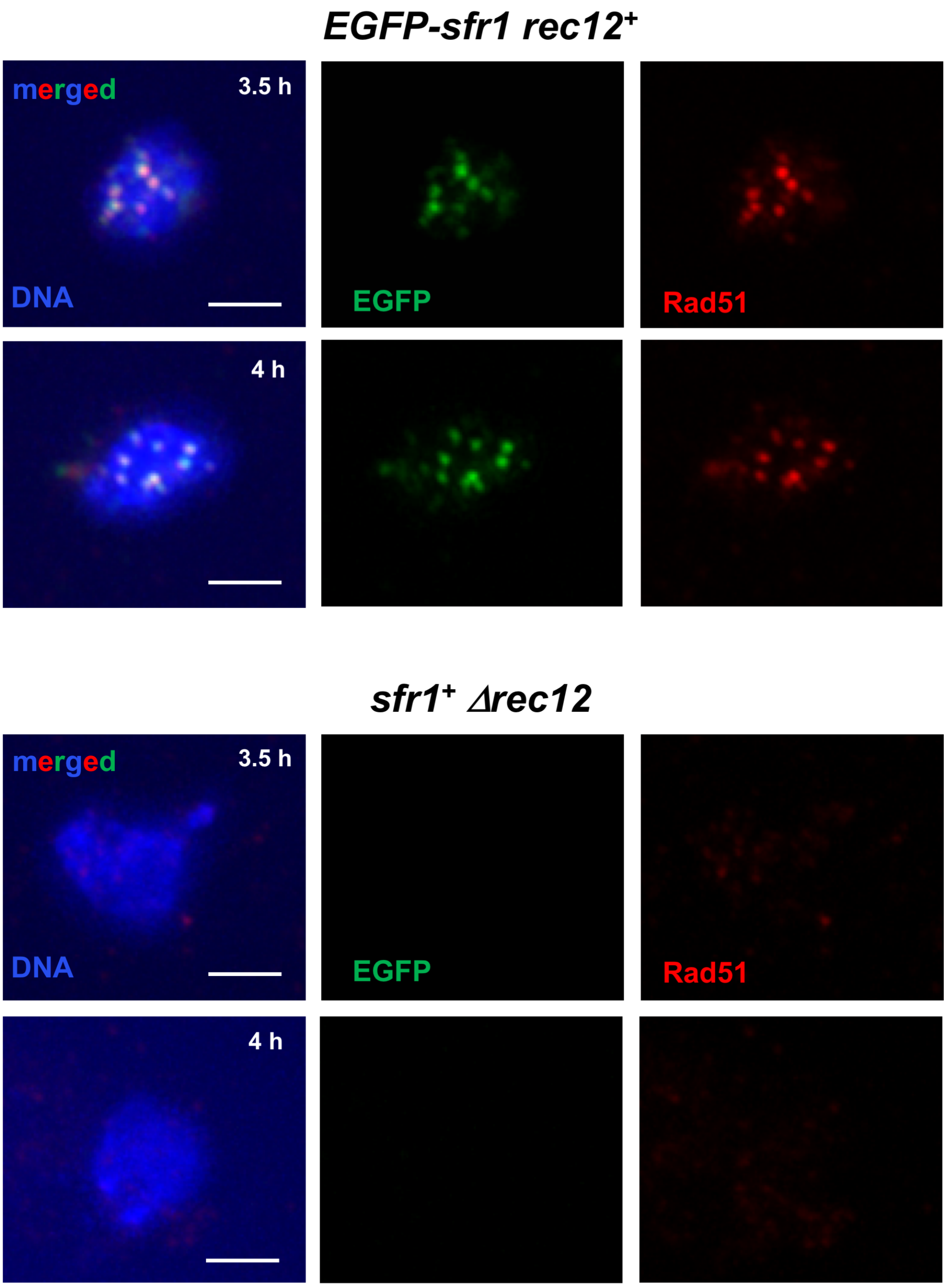

Appendix Figure S7

Appendix Figure S7. Anti-GFP and anti-Rad51 specificity.

pat1-114 EGFP-sfr1 (CMC1649) and *pat1-114 Δrec12* (CMC1901) diploid cells were induced to enter meiosis and collected at different time points during prophase for nuclear spread preparation. Spreads were stained with anti-GFP antibodies (rabbit IgG fraction, A11122 Molecular Probes) for the visualization of EGFP-Sfr1 protein (in green) and with anti-Rad51 antibodies (monoclonal 51RAD01/3C10, Invitrogen) (in red). DAPI staining to visualize DNA is shown in blue. Representative images of nuclei at 3.5 and 4 h after meiotic induction are shown (maximum Z projections). Scale bars correspond to 2 μm.

Strain	Genotype	Used in
**CMC2**	*h⁺ 975*	Fig. 5, Fig. EV4B, Fig. S4B
**CMC319**<br>**CMC1474**	*h⁻ leu1-32*	Fig. 3B, Fig. S1, Fig. S4A
**CMC737**	*h⁺ his5-303*	Fig. 3B, Fig. S1, Fig. S4A
**CMC1074**	*h⁻/h⁻ pat1-114/pat1-114 leu1-32/leu1-32 ade6-M210/ade6-M216*	Fig. 2B
**CMC1322**	*h⁻ ade6-M26 leu1-32::integrant pJK148 (empty)*	Fig. 6B
**CMC1324**	*h⁺ ade6-3049 leu1-32::integrant pJK148 (empty)*	Fig. 6B
**CMC1343**	*h⁻ sfr1::ura5⁺lys7⁺ ura5-14 lys7-2*	Transformation *sfr1* and *EGFP-sfr1* mutants
**CMC1372**	*h⁻ ade6-M26*	Fig. 3C
**CMC1373**	*h⁺ ade6-3049*	Fig. 3C
**CMC1380**	*h⁻ leu1-32 sfr1-7D*	Fig. 3B, Fig. S4A
**CMC1381**	*h⁺ his5-303 sfr1-7D*	Fig. 3B, Fig. S4A
**CMC1408**	*h⁺ his5-303 sfr1::ura5+lys7+ ura5-14 lys7-2*	Fig. 3B, Fig. S4A
**CMC1427**	*h⁻ leu1-32 sfr1::ura5+lys7+ ura5-14 lys7-2*	Fig. 3B, Fig. S4A
**CMC1429**	*h⁻ VL1 ade6-D20 leu1-32::integrant pJK148 (empty)*<br>*(VL1=intg::ade6D5´-hphR-ade6D3´)*	Fig. 6A
**CMC1431**	*h⁺ ade6-D20 leu1-32::integrant pJK148 (empty)*	Fig. 6A
**CMC1434**	*h⁻ VL1 ade6-D20 leu1-32::integrant pJK148 cdc2*<br>*(VL1=intg::ade6D5´-hphR-ade6D3´)*	Fig. 6A
**CMC1436**	*h⁺ ade6-D20 leu1-32::integrant pJK148 cdc2*	Fig. 6A
**CMC1511**	*h⁺ ade6-D20 leu1-32::integrant pJK148 cdc13 (single copy)*	Fig. 6A
**CMC1512**	*h⁻ VL1 ade6-D20 leu1-32::integrant pJK148 cdc13 (single copy)*<br>*(VL1=intg::ade6D5´-hphR-ade6D3´)*	Fig. 6A
**CMC1513**	*h⁺ ade6-D20 leu1-32::integrant pJK148 cdc13 (two copies)*	Fig. 6A
**CMC1514**	*h⁻ VL1 ade6-D20 leu1-32::integrant pJK148 cdc13 (two copies)*<br>*(VL1=intg::ade6D5´-hphR-ade6D3´)*	Fig. 6A
**CMC1515**	*h⁻ ade6-M26 leu1-32::integrant pJK148 cdc2*	Fig. 6B
**CMC1516**	*h⁺ ade6-3049 leu1-32::integrant pJK148 cdc2*	Fig. 6B
**CMC1528**	*h⁺ his5-303 sfr1-7A*	Fig. 3B
**CMC1529**	*h⁻ leu1-32 sfr1-7A*	Fig. 3B
**CMC1534**	*h⁻ ade6-M26 leu1-32::integrant pJK148 cdc13 (single copy)*	Fig. 6B
**CMC1535**	*h⁺ ade6-3049 leu1-32::integrant pJK148 cdc13 (single copy)*	Fig. 6B
**CMC1536**	*h⁻ ade6-M26 leu1-32::integrant pJK148 cdc13 (two copies)*	Fig. 6B

**CMC1537**	*h^+ ade6-3049 leu1-32::integrant pJK148 cdc13 (two copies)*	Fig. 6B
**CMC1629**	*h^+ EGFP-sfr1 his5-303*	Fig. S1
**CMC1630**	*h^- EGFP-sfr1 leu1-32*	Fig. S1
**CMC1649**	*h^-/h^- pat1-114/pat1-114 leu1-32/leu1-32 ade6-M210/ade6-M216 EGFP-sfr1/EGFP-sfr1*	Fig. 1A-B and D, Fig. 2B-C, Fig. 4, Fig. EV2, Fig. EV3, Fig. EV4A, Fig. S2, Fig. S3C, Fig. S4C
**CMC1660**	*h^-/h^- pat1-114/pat1-114 leu1-32/leu1-32 ade6-M210/ade6-M216 EGFP-sfr1/EGFP-sfr1 cdc2-asM17/cdc2-asM17*	Fig. 2A
**CMC1704**	*h^- EGFP-sfr1-7A*	Fig. 5, Fig. EV4B
**CMC1712**	*h^- EGFP-sfr1-7D*	Fig. 5, Fig. EV4B
**CMC1716**	*h^- sfr1-7D*	Fig. S3A-B
**CMC1733**	*h^-/h^- pat1-114/pat1-114 leu1-32/leu1-32 ade6-M210/ade6-M216 EGFP-sfr1-7A/EGFP-sfr1-7A*	Fig. 1D, Fig. 4, Fig. EV2, Fig. EV3, Fig. EV4A, Fig. S2, Fig. S3C
**CMC1735**	*h^+ ura4-D18 rhp51ECFP-$ura4^+$-rhp51*	Fig. S3A-B
**CMC1738**	*h^+ ura4-D18 rhp51ECFP-$ura4^+$-rhp51 sfr1-7D*	Fig. S3A-B
**CMC1743**	*h^- EGFP-sfr1-WI*	Fig. S4B
**CMC1756**	*h^-/h^- pat1-114/pat1-114 leu1-32/leu1-32 ade6-M210/ade6-M216 EGFP-sfr1-7D/EGFP-sfr1-7D*	Fig. 4, Fig. EV2, Fig. EV3, Fig. EV4A, Fig. S2, Fig. S3C
**CMC1765**	*h^- sfr1-WI*	Fig. S3A-B
**CMC1766**	*h^+ his5-303 sfr1-WI*	Fig. S4A
**CMC1767**	*h^- leu1-32 sfr1-WI*	Fig. S4A
**CMC1769**	*h-/h- pat1-114/pat1-114 leu1-32/leu1-32 ade6-M210/ade6-M216 EGFP-sfr1-WI/EGFP-sfr1-WI*	Fig. 4A-B, Fig. EV2, Fig. EV3A, Fig. S2, Fig. S3C, Fig. S4C
**CMC1776**	*h^+ ura4-D18 rhp51ECFP-$ura4^+$-rhp51 sfr1-WI*	Fig. S3A-B
**CMC1788**	*h^- EGFP-sfr1*	Fig. 5, Fig. EV4B, Fig. S4B
**CMC1789**	*h^+ sfr1-WI*	Fig. S4B
**CMC1790**	*h^+ sfr1-7D*	Fig. 5, Fig. EV4B
**CMC1791**	*h^+ sfr1-7A*	Fig. 5, Fig. EV4B
**CMC1794**	*h^- sfr1-7A*	Fig. S3A-B
**CMC1802**	*h^+ ura4-D18 rhp51ECFP-$ura4^+$-rhp51 sfr1-7A*	Fig. S3A-B.
**CMC1808**	*h^+ rec12::kanMX6*	Fig. 5
**CMC1809**	*h^- rec12::kanMX6 EGFP-sfr1*	Fig. 5
**CMC1826**	*h- 972*	Fig. S3A-B
**CMC1843**	*h^{90} $lys1^+$::LacO $his7^+$::LacI-GFP*	Fig. 7

**CMC1845**	*h^90 lys1^+::LacO his7^+::LacI-GFP sfr1-11::hphMX4*	Fig. 7
**CMC1847**	*h^90 lys1^+::LacO his7^+::LacI-GFP sfr1-7A*	Fig. 7
**CMC1848**	*h^90 lys1^+::LacO his7^+::LacI-GFP sfr1-7D*	Fig. 7
**CMC1859**	*h^90 lys1^+::LacO his7^+::LacI-GFP dbl2::natMX4 sfr1-7D*	Fig. 7
**CMC1860**	*h^90 lys1^+::LacO his7^+::LacI-GFP dbl2::natMX4*	Fig. 7
**CMC1861**	*h^90 lys1^+::LacO his7^+::LacI-GFP dbl2::natMX4 sfr1-11::hphMX4*	Fig. 7
**CMC1862**	*h^90 lys1^+::LacO his7^+::LacI-GFP dbl2::natMX4 sfr1-7A*	Fig. 7
**CMC1881**	*h^+ ade6-3049 sfr1-11::hphMX4*	Fig. 3C
**CMC1883**	*h^- ade6-M26 sfr1-11::hphMX4*	Fig. 3C
**CMC1884**	*h^+ ade6-3049 sfr1-7A*	Fig. 3C
**CMC1886**	*h^- ade6-M26 sfr1-7A*	Fig. 3C
**CMC1887**	*h^+ ade6-3049 sfr1-7D*	Fig. 3C
**CMC1888**	*h^- ade6-M26 sfr1-7D*	Fig. 3C
**CMC1894**	*h^+ ura4-D18 rhp51ECFP-ura4^+-rhp51 rec12::kanMX6*	Fig. S3A-B
**CMC1896**	*h^+ rec12::kanMX6*	Fig. S3A-B
**CMC1901**	*h-/h- pat1-114/pat1-114 leu1-32/leu1-32 ade6-M210/ade6-M216 rec12::kanMX6/rec12::kanMX6*	Fig. S7

Appendix Table S1. ***S. pombe*** **strains**

Alleles other than commonly used auxotrophies and mating type are *pat1-114* (Iino & Yamamoto, 1985), *rec12::KanMX6* (Martín-Castellanos *et al*, 2005), *EGFP-sfr1* (Akamatsu *et al*, 2007), *rhp51ECFP-ura4⁺-rhp51* (Akamatsu *et al*, 2007), *dbl2::natMX4* (Gregan *et al*, 2005), *sfr1-11::hphMX4* (Lorenz *et al*, 2014) and *sfr1::ura5-lys7* (complete deletion generated in this study). VL1 system (*intg::ade6D5´-hphR-ade6D3´*) is described in (Latypov *et al*, 2010). *pJK148 integrants* (empty, *cdc2* and *cdc13*) are described in (Bustamante-Jaramillo *et al*, 2019).

Appendix References

Akamatsu Y, Jasin M (2010) Role for the mammalian Swi5-Sfr1 complex in DNA strand break repair through homologous recombination. *PLoS Genet* 6: e1001160

Akamatsu Y, Tsutsui Y, Morishita T, Siddique MS, Kurokawa Y, Ikeguchi M, Yamao F, Arcangioli B, Iwasaki H (2007) Fission yeast Swi5/Sfr1 and Rhp55/Rhp57 differentially regulate Rhp51-dependent recombination outcomes. *EMBO J* 26: 1352-1362

Argunhan B, Sakakura M, Afshar N, Kurihara M, Ito K, Maki T, Kanamaru S, Murayama Y, Tsubouchi H, Takahashi M *et al* (2020) Cooperative interactions facilitate stimulation of Rad51 by the Swi5-Sfr1 auxiliary factor complex. *Elife* 9

Bernhofer M, Goldberg T, Wolf S, Ahmed M, Zaugg J, Boden M, Rost B (2018) NLSdb-major update for database of nuclear localization signals and nuclear export signals. *Nucleic Acids Res* 46: D503-D508

Bustamante-Jaramillo LF, Ramos C, Alonso L, Sesmero A, Segurado M, Martin-Castellanos C (2019) CDK contribution to DSB formation and recombination in fission yeast meiosis. *PLoS Genet* 15: e1007876

Gregan J, Rabitsch PK, Sakem B, Csutak O, Latypov V, Lehmann E, Kohli J, Nasmyth K (2005) Novel genes required for meiotic chromosome segregation are identified by a high-throughput knockout screen in fission yeast. *Curr Biol* 15: 1663-1669

Iino Y, Yamamoto M (1985) Negative control for the initiation of meiosis in Schizosaccharomyces pombe. *Proc Natl Acad Sci U S A* 82: 2447-2451

Martin-Castellanos C, Blanco M, Rozalen AE, Perez-Hidalgo L, Garcia AI, Conde F, Mata J, Ellermeier C, Davis L, San-Segundo P *et al* (2005) A large-scale screen in S. pombe identifies seven novel genes required for critical meiotic events. *Curr Biol* 15: 2056-2062

Nguyen Ba AN, Pogoutse A, Provart N, Moses AM (2009) NLStradamus: a simple Hidden Markov Model for nuclear localization signal prediction. *BMC Bioinformatics* 10: 202

Latypov V, Rothenberg M, Lorenz A, Octobre G, Csutak O, Lehmann E, Loidl J, Kohli J (2010) Roles of Hop1 and Mek1 in meiotic chromosome pairing and recombination partner choice in Schizosaccharomyces pombe. *Mol Cell Biol* 30: 1570-1581

Lorenz A, Mehats A, Osman F, Whitby MC (2014) Rad51/Dmc1 paralogs and mediators oppose DNA helicases to limit hybrid DNA formation and promote crossovers during meiotic recombination. *Nucleic Acids Res* 42: 13723-13735
