# Supplementary material for: CDK phosphorylation of Sfr1 downregulates Rad51 function in late-meiotic homolog invasions
Source: EMBO J. 2024 Aug 22;43(19):4356–83. doi: 10.1038/s44318-024-00205-2 (PMC11445502; doi:10.1038/s44318-024-00205-2)
Supplement: Supplementary file 8 — Movie EV2 [file 44318_2024_205_MOESM8_ESM.zip › Movie EV2/Movie EV2 Legend.docx]

**Movie EV2.** **Time lapse of *EGFP-sfr1 Δrec12* zygote.**

Time lapse experiment showing EGFP-Sfr1 in the absence of meiotic DSBs (*Δrec12* mutant). Zygotes were obtained in crosses of *h^-^ EGFP-sfr1* *Δrec12* (CMC1809) X *h^+^* *Δrec12* (CMC1808) strains. Images were taken every 5 minutes; frames correspond to maximal projections (9 Z sections, 0.5 μm step size). Scale bar corresponds to 5 μm. Related to Figure 5.
