# Supplementary material for: CDK phosphorylation of Sfr1 downregulates Rad51 function in late-meiotic homolog invasions
Source: EMBO J. 2024 Aug 22;43(19):4356–83. doi: 10.1038/s44318-024-00205-2 (PMC11445502; doi:10.1038/s44318-024-00205-2)
Supplement: Supplementary file 10 — Movie EV4 [file 44318_2024_205_MOESM10_ESM.zip › Movie EV4/Movie EV4 Legend.docx]

**Movie EV4.** **Time lapse of *EGFP-sfr1-7D* zygote.**

Time lapse experiment showing EGFP-Sfr1-7D. Zygotes were obtained in crosses of *h^-^ EGFP-sfr1*-7D (CMC1712) X *h^+^* *sfr1-7D* (CMC1790) strains. Images were taken every 5 minutes; frames correspond to maximal projections (11 Z sections, 0.5 μm step size). Scale bar corresponds to 5 μm. Related to Figure 5.
