# Supplementary material for: CDK phosphorylation of Sfr1 downregulates Rad51 function in late-meiotic homolog invasions
Source: EMBO J. 2024 Aug 22;43(19):4356–83. doi: 10.1038/s44318-024-00205-2 (PMC11445502; doi:10.1038/s44318-024-00205-2)
Supplement: Supplementary file 12 — Movie EV6 [file 44318_2024_205_MOESM12_ESM.zip › Movie EV6/Movie EV6 Legend.docx]

**Movie EV6.** **Time lapse of *rad51-ECFP* *Δrec12* zygote.**

Time lapse experiment showing Rad51-ECFP in the absence of meiotic DSBs (*Δrec12* mutant). Zygotes were obtained in crosses of *h^+^ rad51-ECFP-ura4^+^-rad51* *Δrec12* (CMC1894) X *h^-^* *Δrec12* (CMC1896) strains. Images were taken every 10 minutes; frames correspond to maximal projections (11 Z sections, 0.5 μm step size). Scale bar corresponds to 5 μm. Related to Appendix Figure S3.
