# Supplementary material for: CDK phosphorylation of Sfr1 downregulates Rad51 function in late-meiotic homolog invasions
Source: EMBO J. 2024 Aug 22;43(19):4356–83. doi: 10.1038/s44318-024-00205-2 (PMC11445502; doi:10.1038/s44318-024-00205-2)
Supplement: Supplementary file 13 — Movie EV7 [file 44318_2024_205_MOESM13_ESM.zip › Movie EV7/Movie EV7 Legend.docx]

**Movie EV7.** **Time lapse of *rad51-ECFP* *sfr1-7A* zygote.**

Time lapse experiment showing Rad51-ECFP in the *sfr1-7A* mutant. Zygotes were obtained in crosses of *h^+^ rad51-ECFP-ura4^+^-rad51* *sfr1-7A* (CMC1802) X *h^-^* *sfr1-7A* (CMC1794) strains. Images were taken every 10 minutes; frames correspond to maximal projections (11 Z sections, 0.5 μm step size). Scale bar corresponds to 5 μm. Related to Appendix Figure S3.
