# Supplementary figures and images for: CDK phosphorylation of Sfr1 downregulates Rad51 function in late-meiotic homolog invasions
Source: EMBO J. 2024 Aug 22;43(19):4356–83. doi: 10.1038/s44318-024-00205-2 (PMC11445502; doi:10.1038/s44318-024-00205-2)

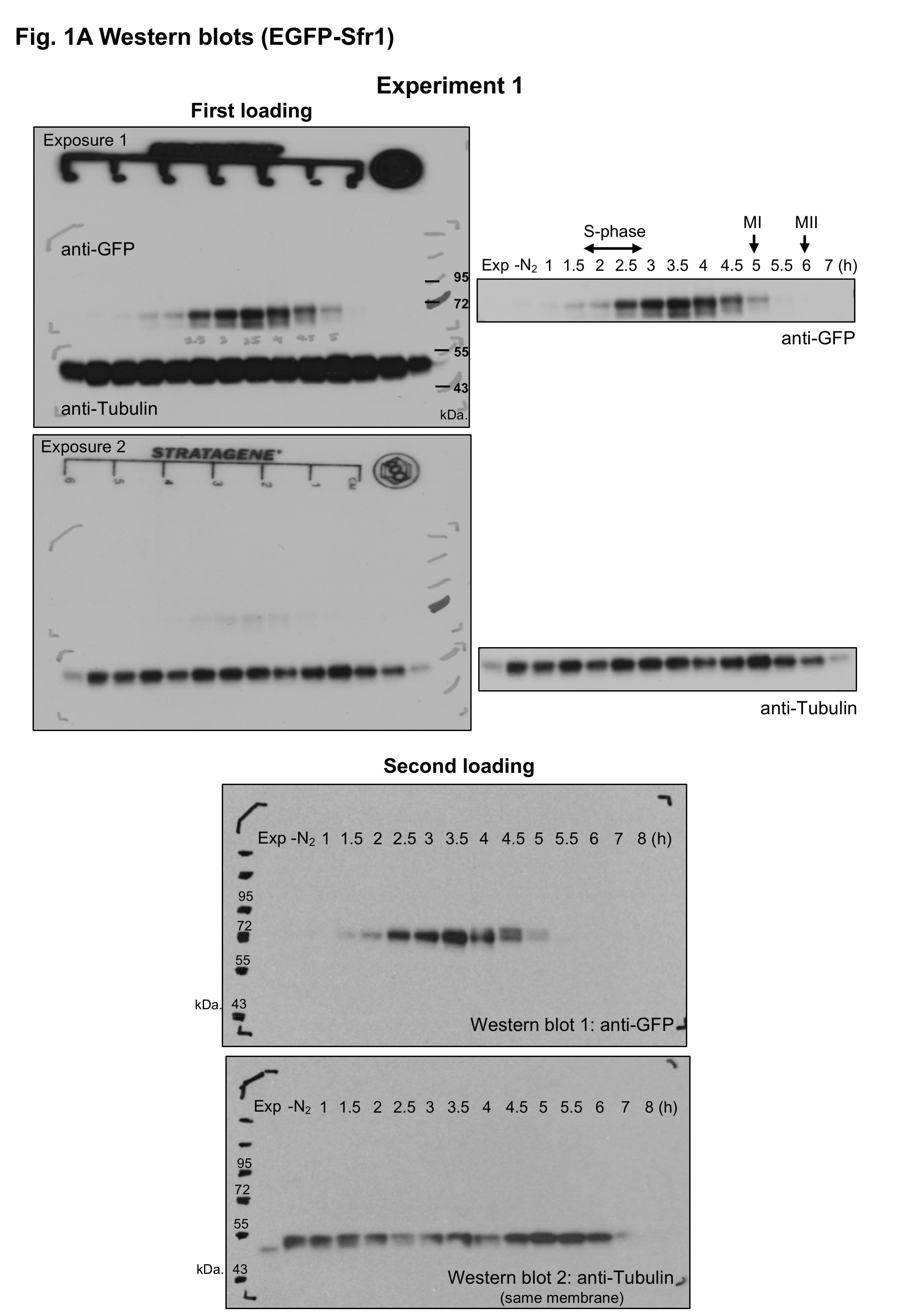

Supplement: Supplementary file 16 — Source data Fig. 1 [file 44318_2024_205_MOESM16_ESM.zip › Figure 1 Source Data/1A/1A Western blots/Experiment 1.tiff]

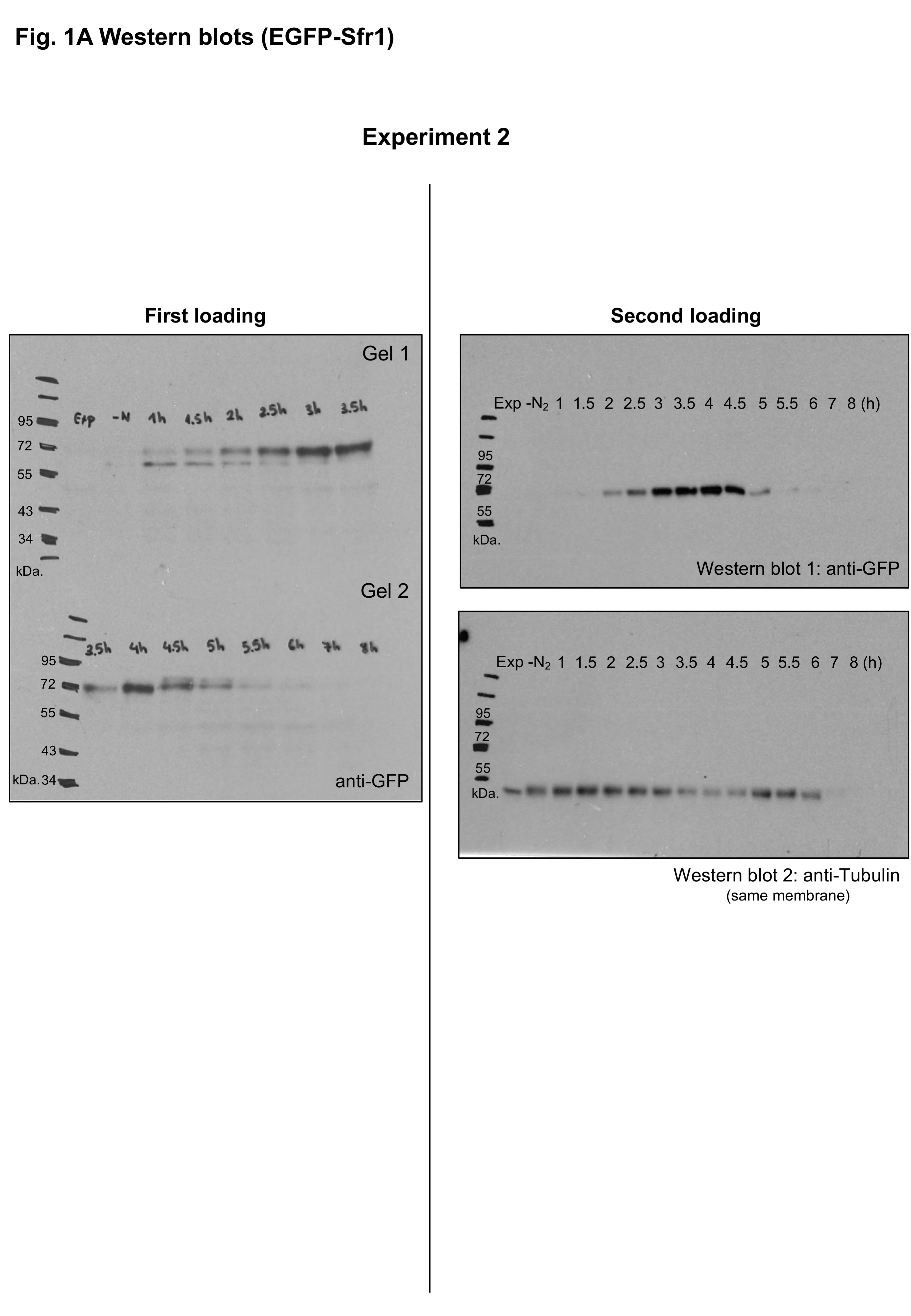

Supplement: Supplementary file 16 — Source data Fig. 1 [file 44318_2024_205_MOESM16_ESM.zip › Figure 1 Source Data/1A/1A Western blots/Experiment 2.tiff]

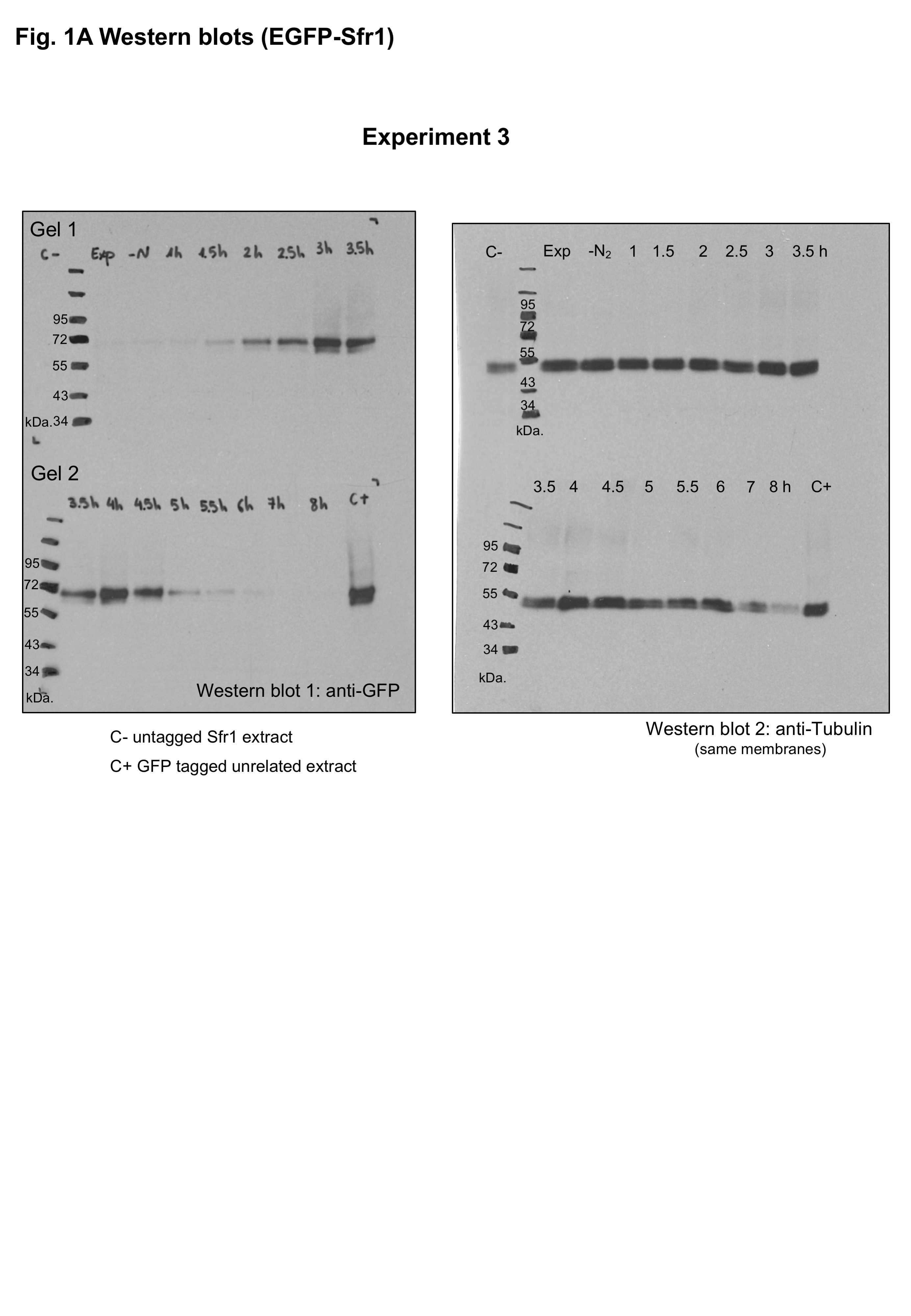

Supplement: Supplementary file 16 — Source data Fig. 1 [file 44318_2024_205_MOESM16_ESM.zip › Figure 1 Source Data/1A/1A Western blots/Experiment 3.tiff]

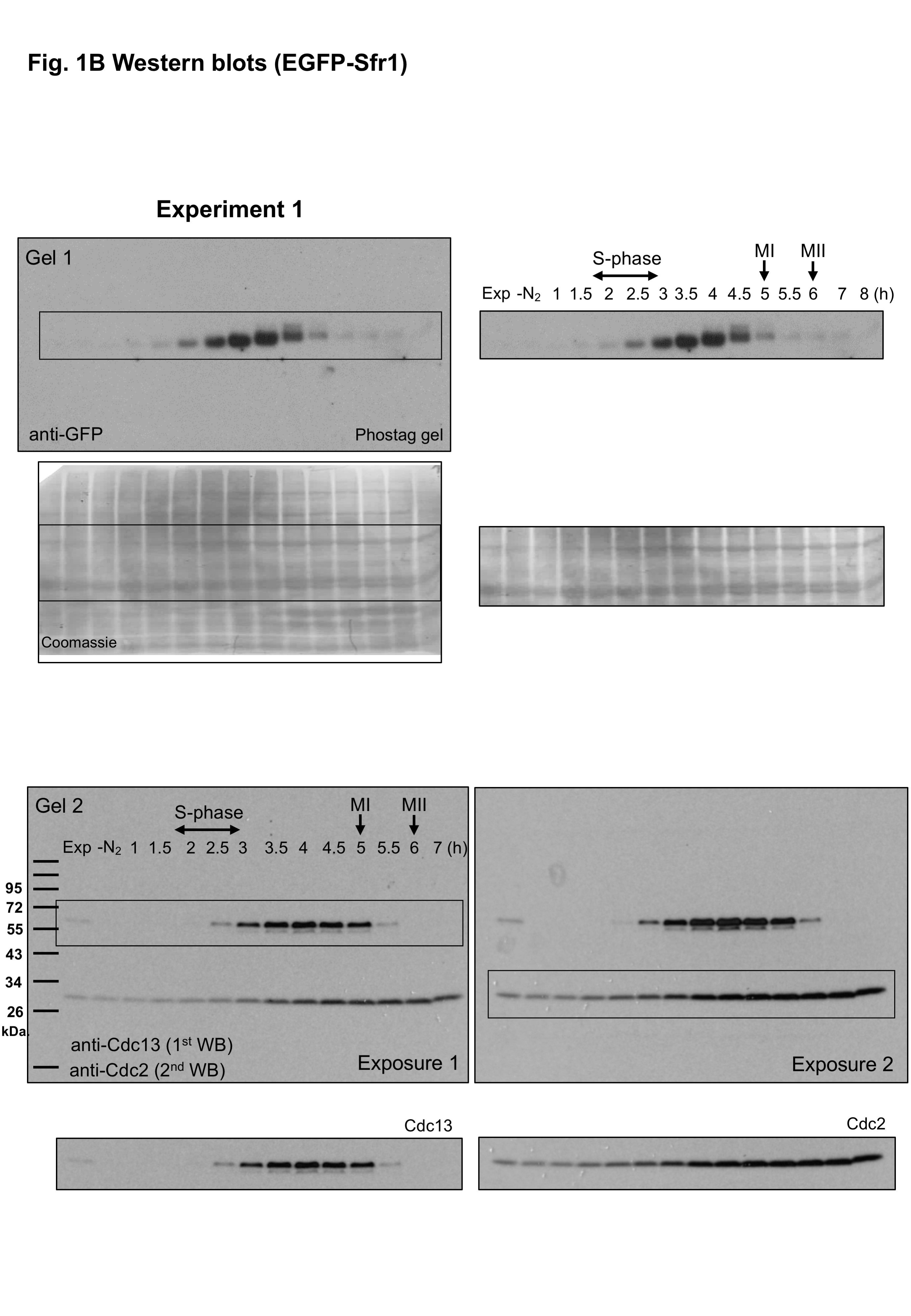

Supplement: Supplementary file 16 — Source data Fig. 1 [file 44318_2024_205_MOESM16_ESM.zip › Figure 1 Source Data/1B/1B Western blots/Experiment 1.tiff]

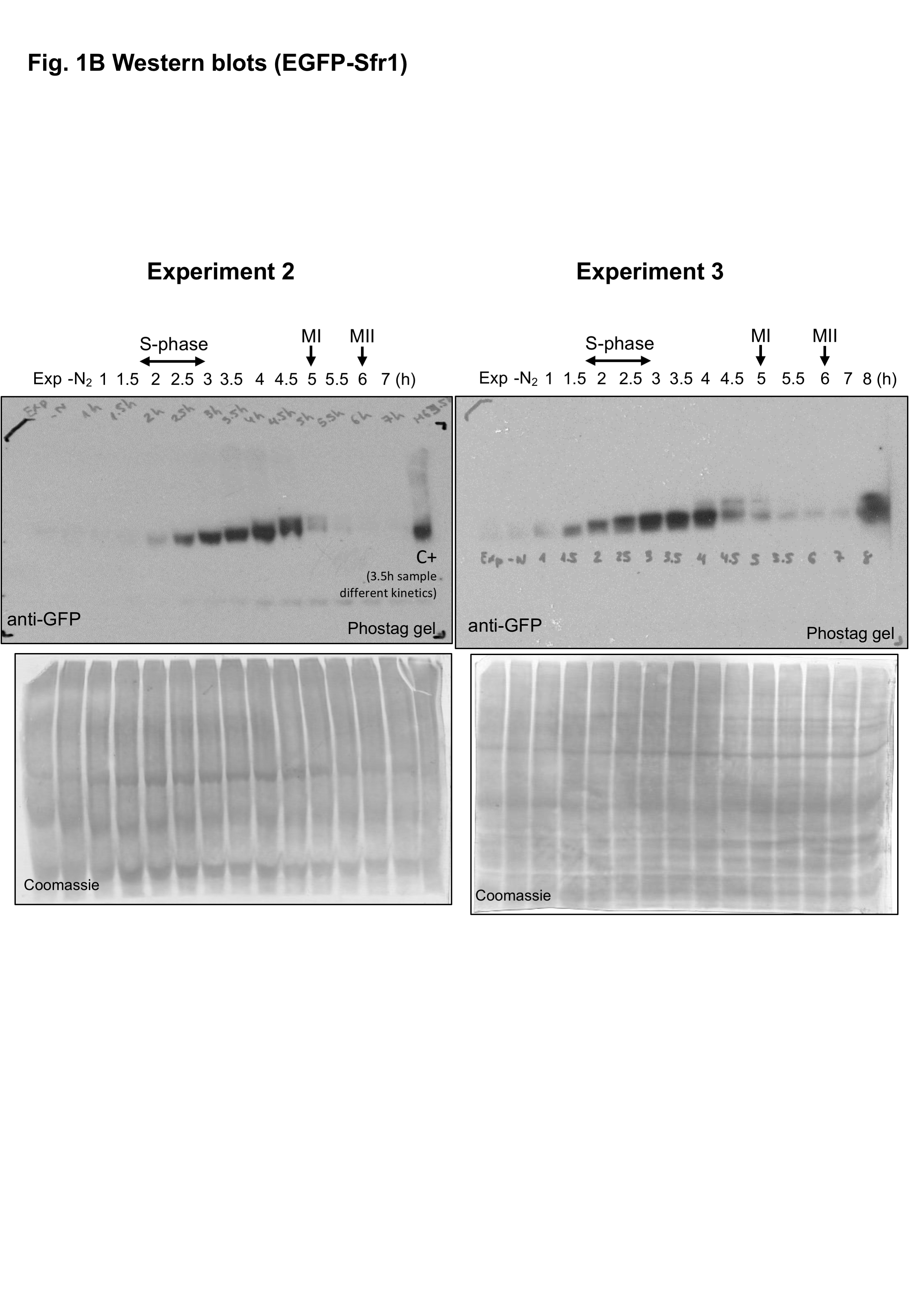

Supplement: Supplementary file 16 — Source data Fig. 1 [file 44318_2024_205_MOESM16_ESM.zip › Figure 1 Source Data/1B/1B Western blots/Experiment 2 and 3.tiff]

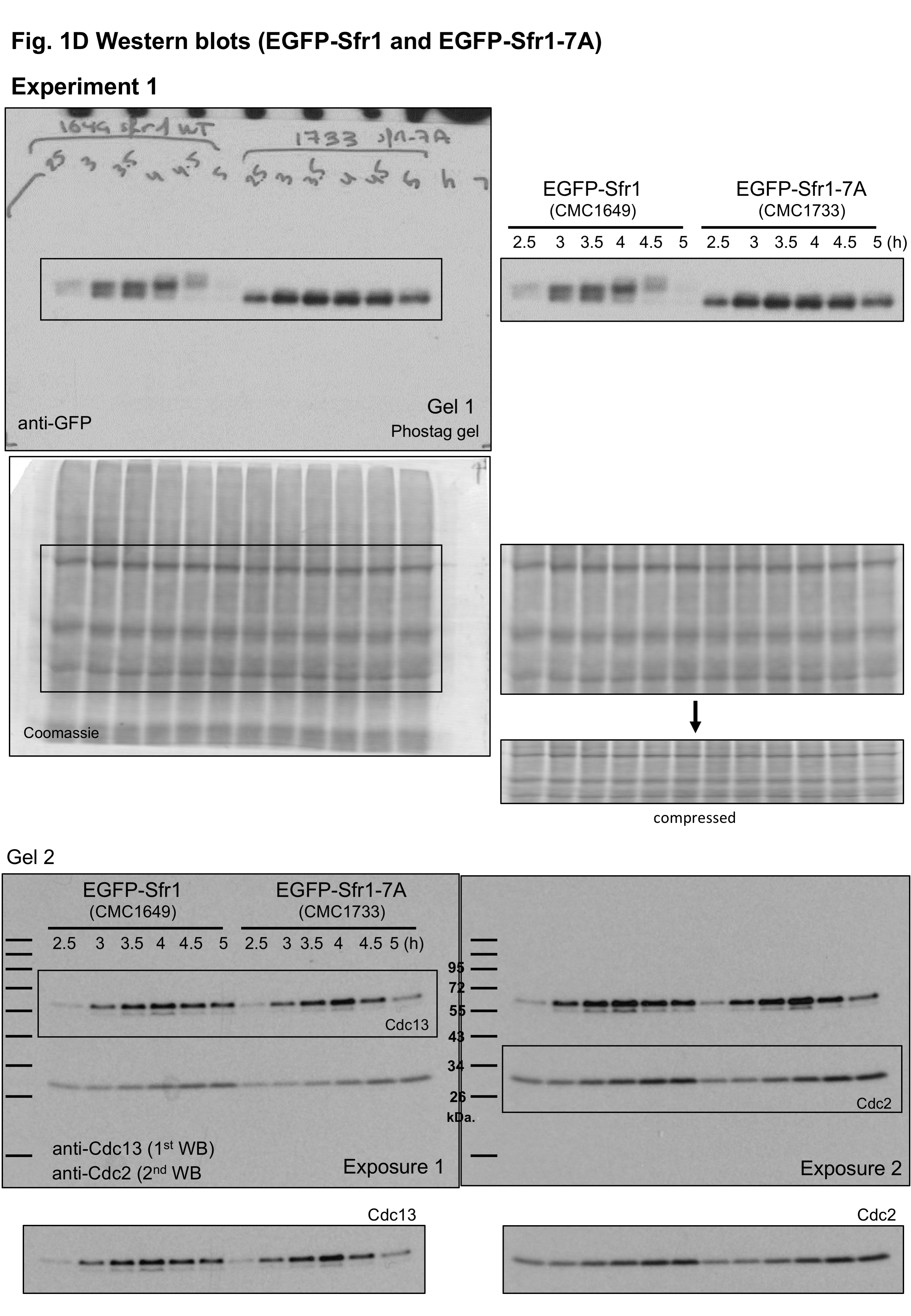

Supplement: Supplementary file 16 — Source data Fig. 1 [file 44318_2024_205_MOESM16_ESM.zip › Figure 1 Source Data/1D/1D Western blots/Experiment 1.tiff]

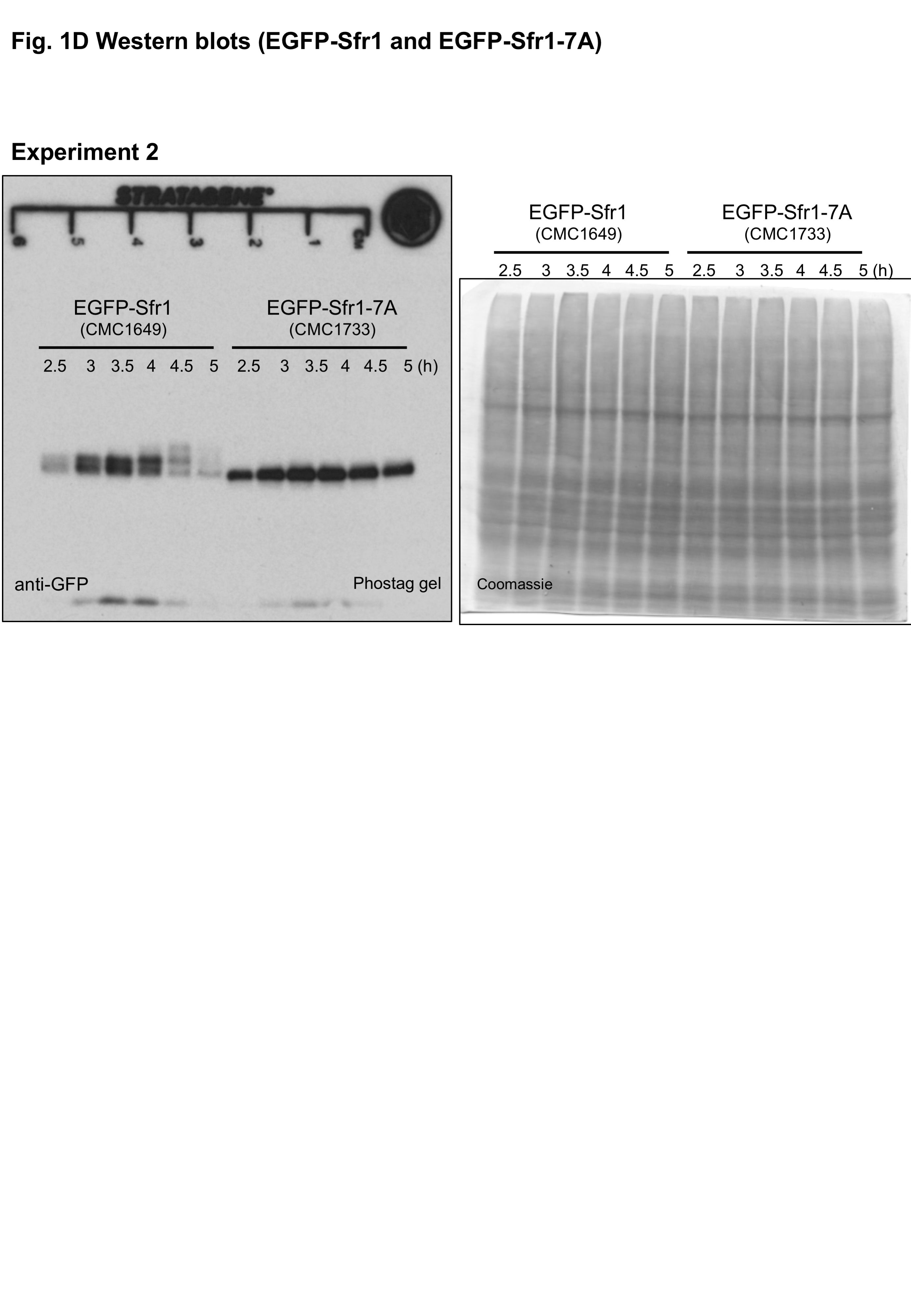

Supplement: Supplementary file 16 — Source data Fig. 1 [file 44318_2024_205_MOESM16_ESM.zip › Figure 1 Source Data/1D/1D Western blots/Experiment 2.tiff]

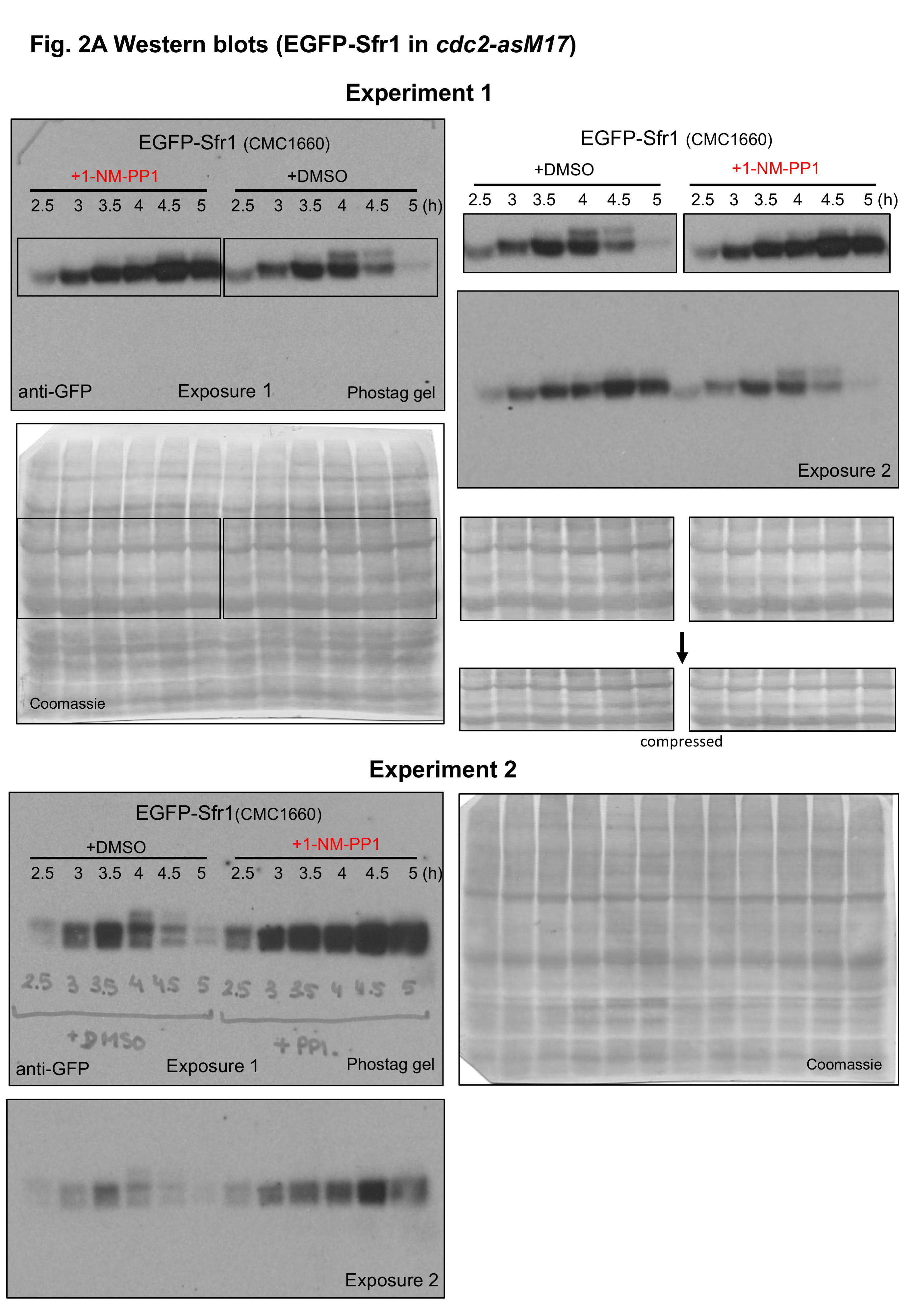

Supplement: Supplementary file 17 — Source data Fig. 2 [file 44318_2024_205_MOESM17_ESM.zip › Figure 2 Source Data/2A/2A Western blots/Experiment 1 and 2.tiff]

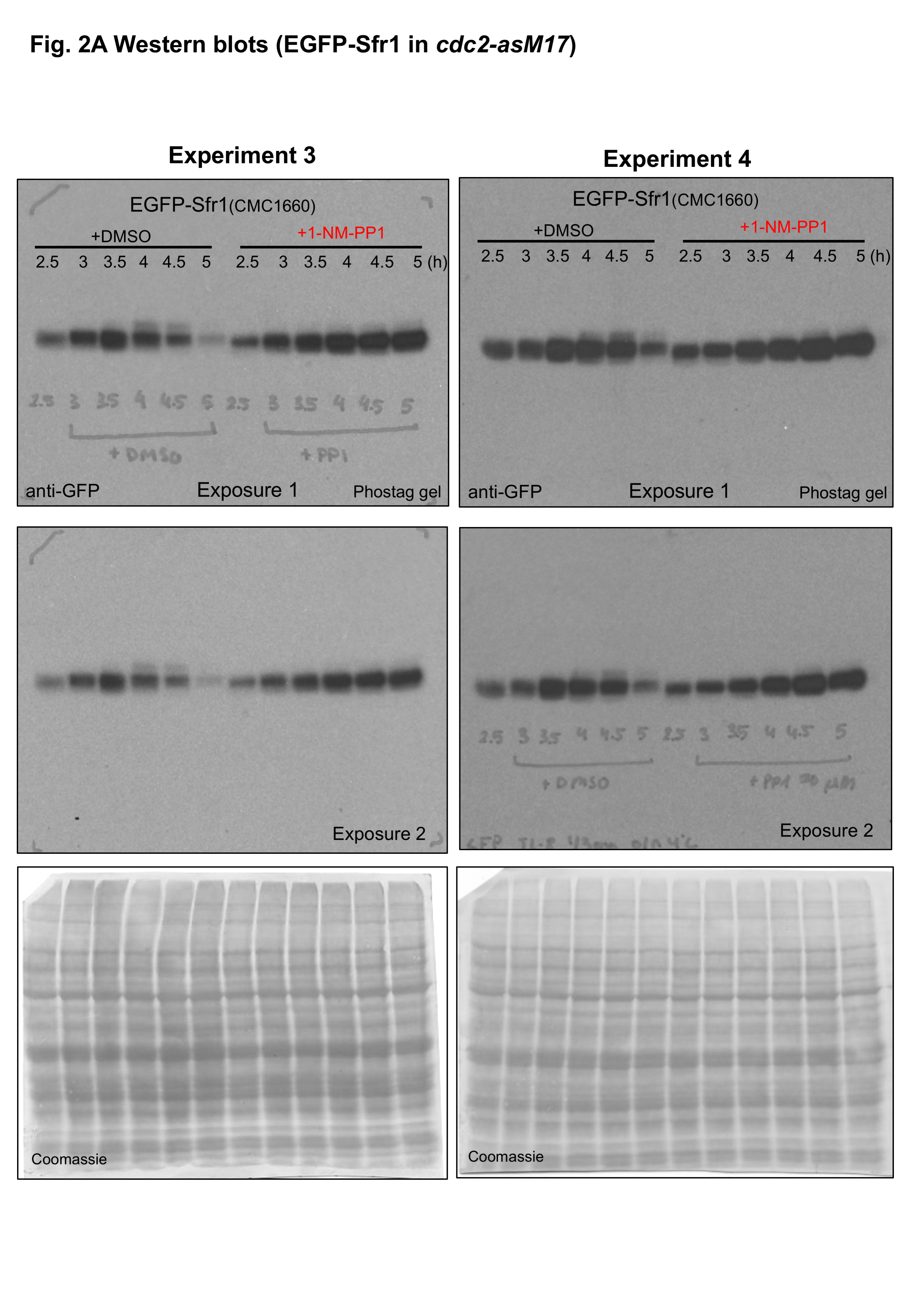

Supplement: Supplementary file 17 — Source data Fig. 2 [file 44318_2024_205_MOESM17_ESM.zip › Figure 2 Source Data/2A/2A Western blots/Experiment 3 and 4.tiff]

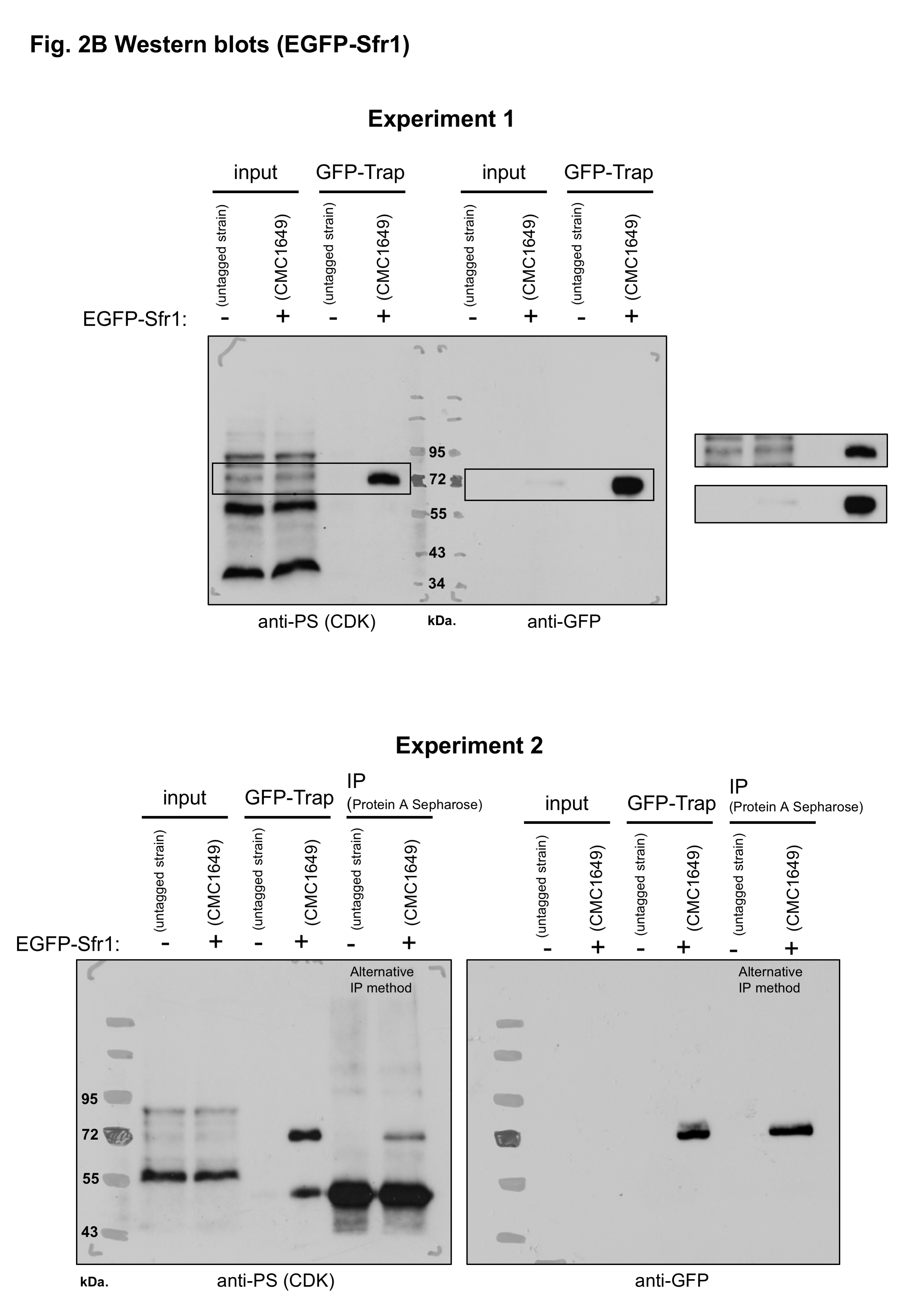

Supplement: Supplementary file 17 — Source data Fig. 2 [file 44318_2024_205_MOESM17_ESM.zip › Figure 2 Source Data/2B/2B Western blots/Experiment 1 and 2.tiff]

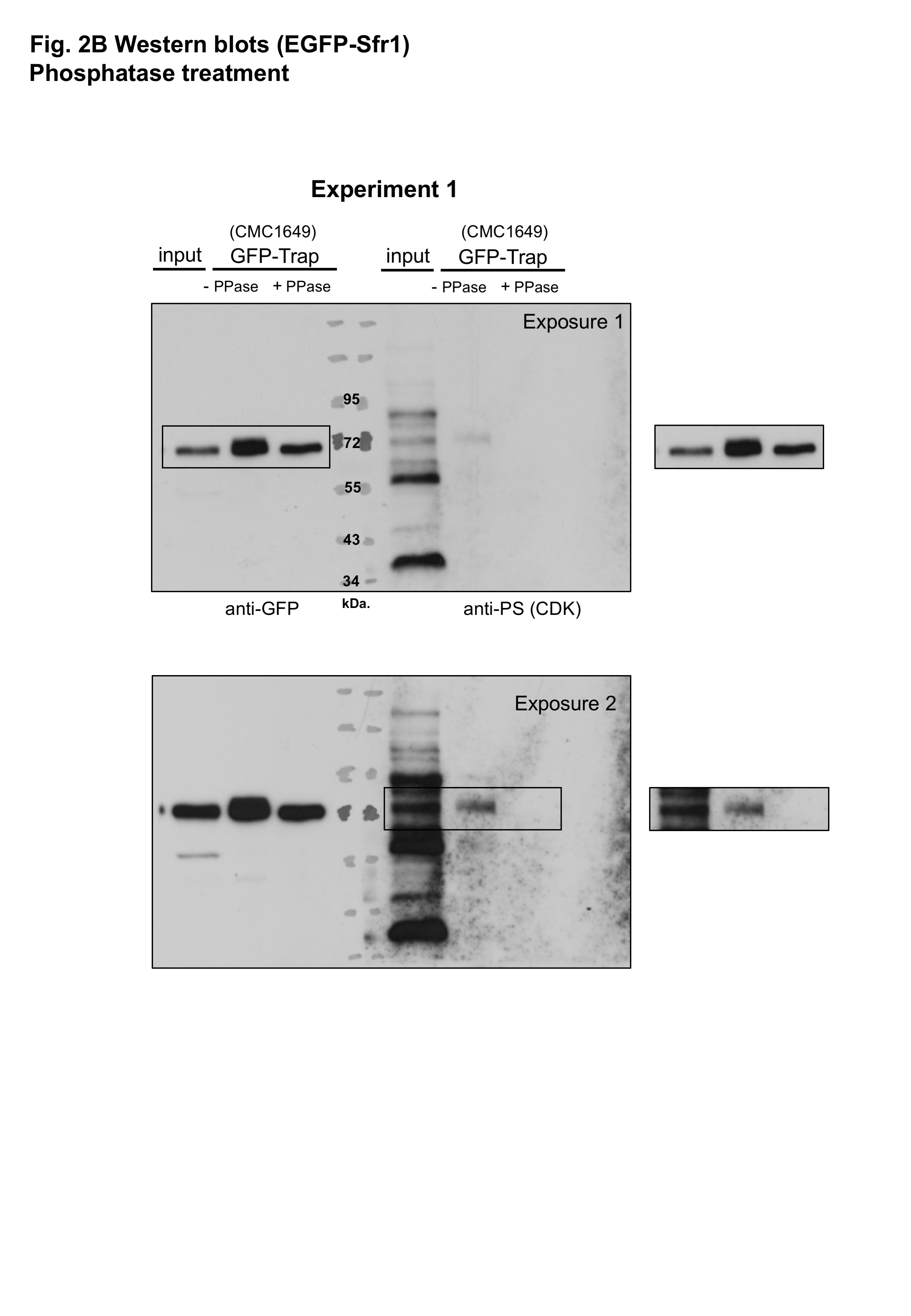

Supplement: Supplementary file 17 — Source data Fig. 2 [file 44318_2024_205_MOESM17_ESM.zip › Figure 2 Source Data/2B/2B Western blots/PPase Experiment 1.tiff]

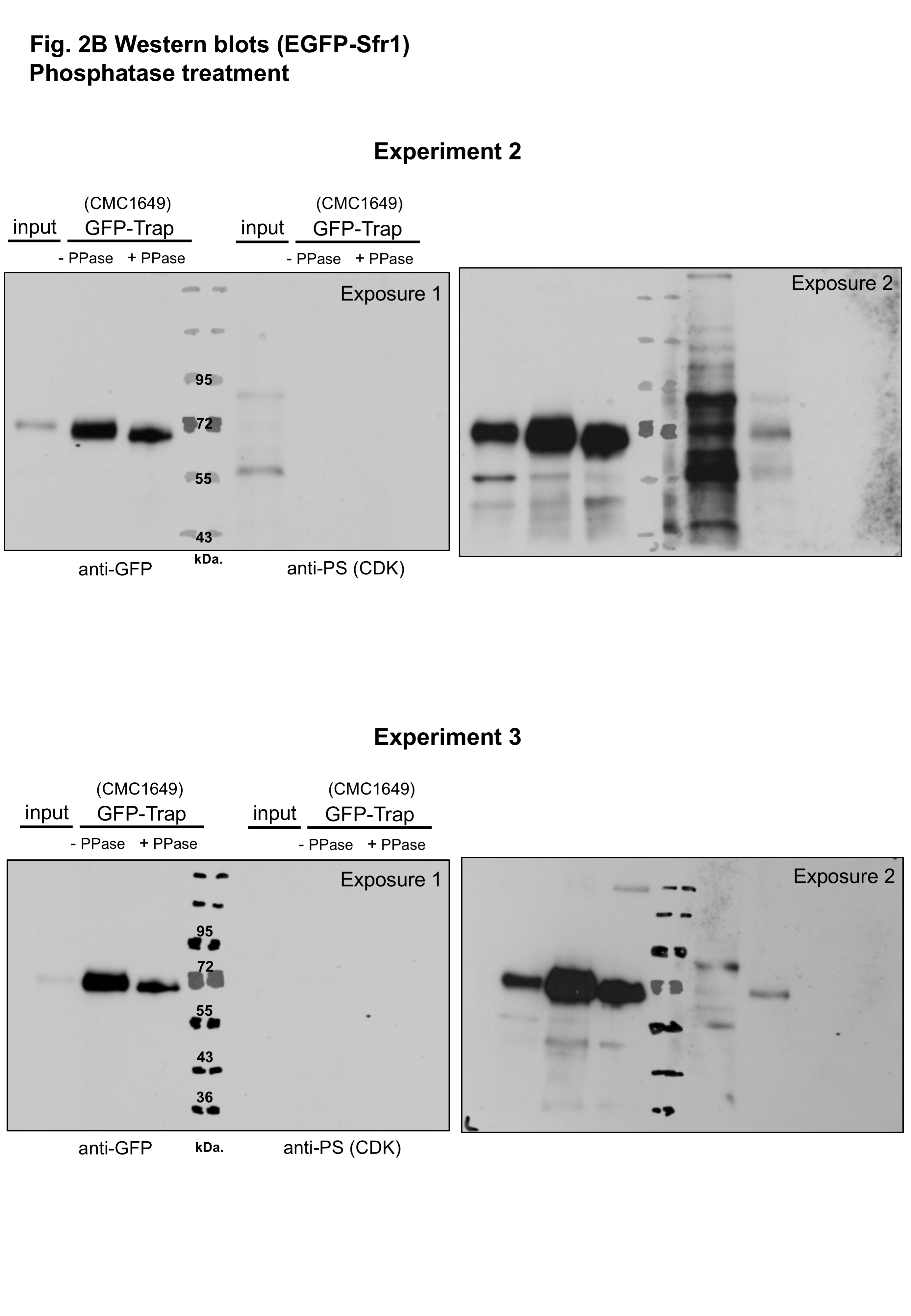

Supplement: Supplementary file 17 — Source data Fig. 2 [file 44318_2024_205_MOESM17_ESM.zip › Figure 2 Source Data/2B/2B Western blots/PPase Experiment 2 and 3.tiff]

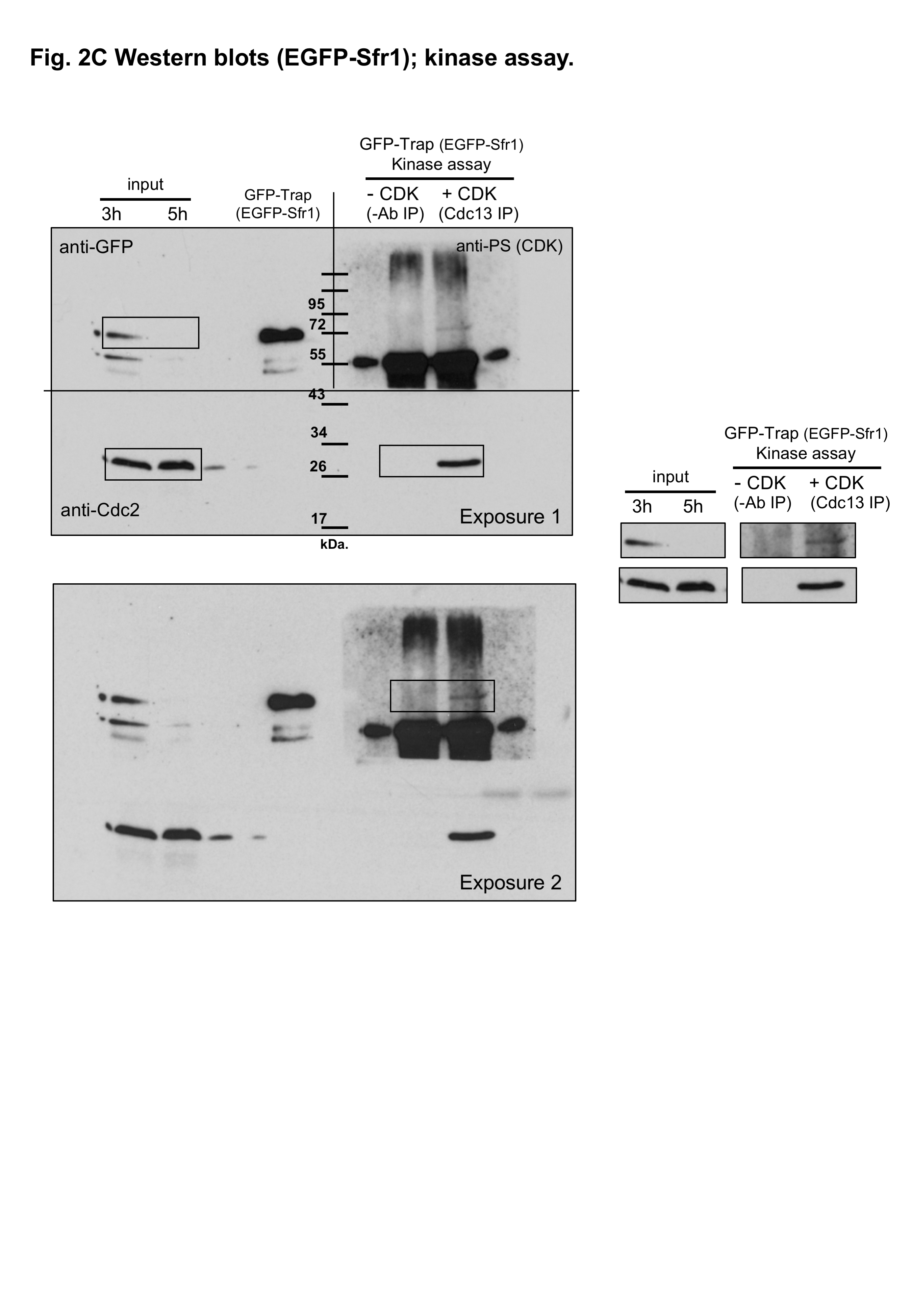

Supplement: Supplementary file 17 — Source data Fig. 2 [file 44318_2024_205_MOESM17_ESM.zip › Figure 2 Source Data/2C/2C Western blots/Kinase assay.tiff]

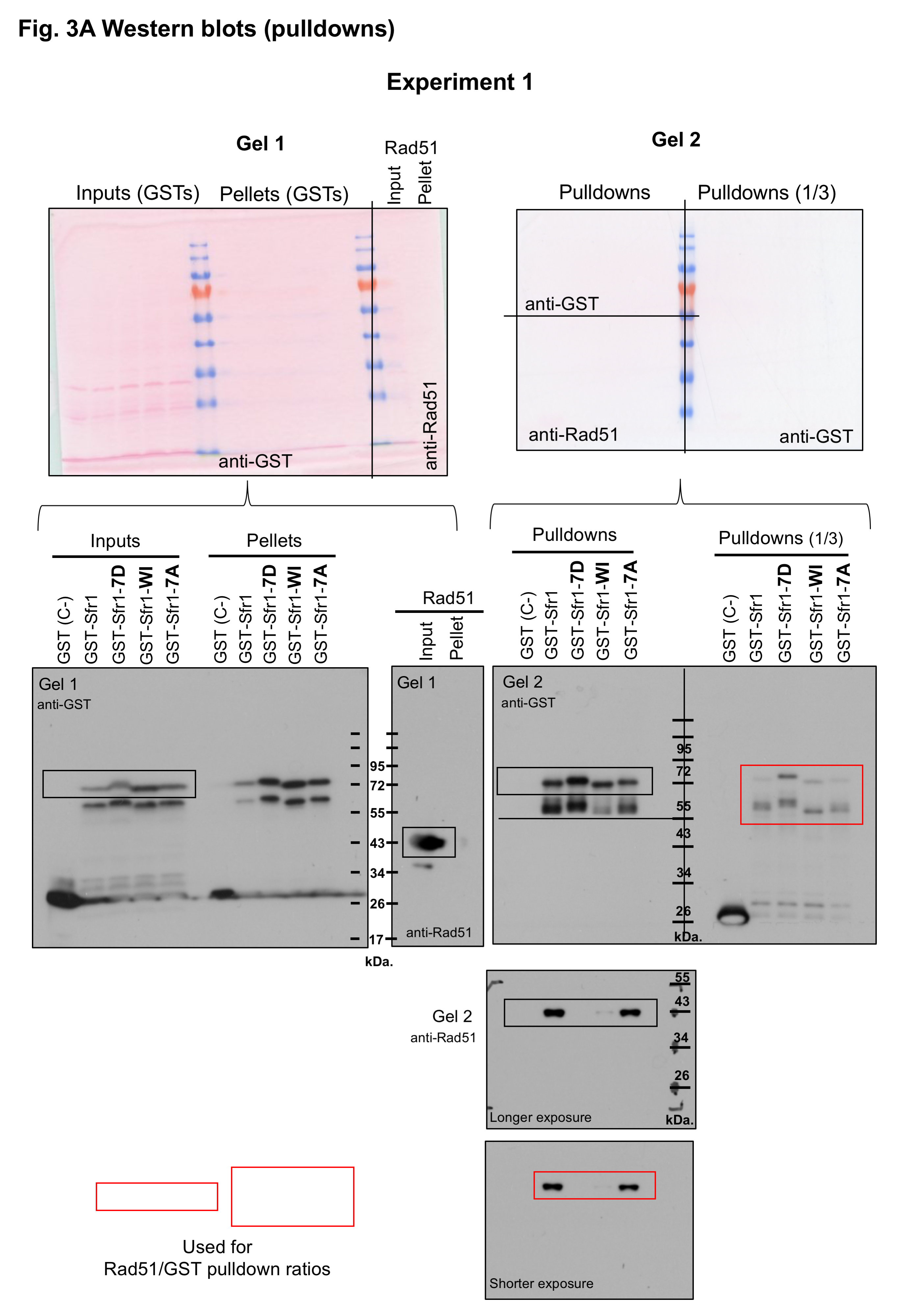

Supplement: Supplementary file 18 — Source data Fig. 3 [file 44318_2024_205_MOESM18_ESM.zip › Figure 3 Source Data/3A/3A Western blots/Experiment 1.tiff]

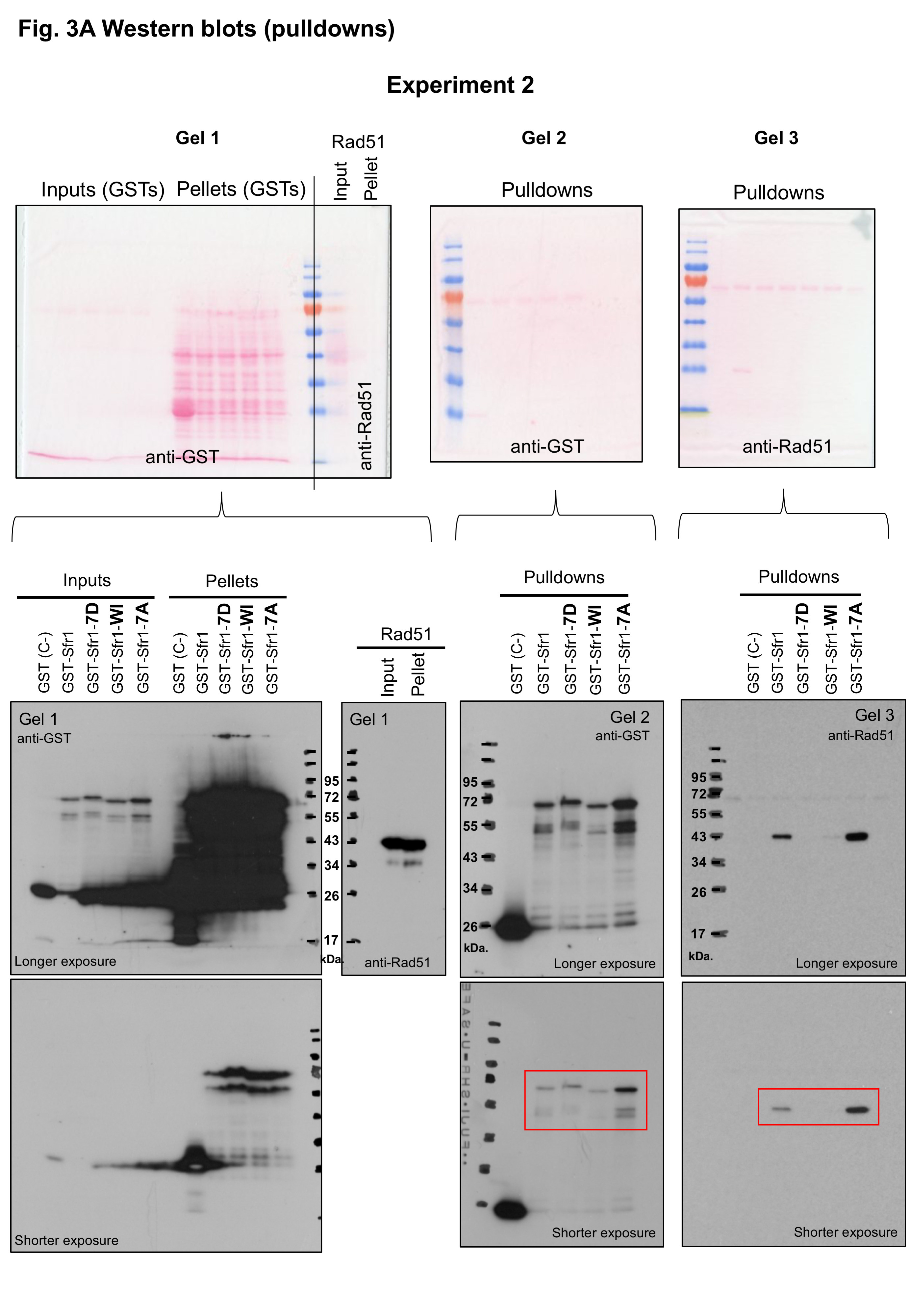

Supplement: Supplementary file 18 — Source data Fig. 3 [file 44318_2024_205_MOESM18_ESM.zip › Figure 3 Source Data/3A/3A Western blots/Experiment 2.tiff]

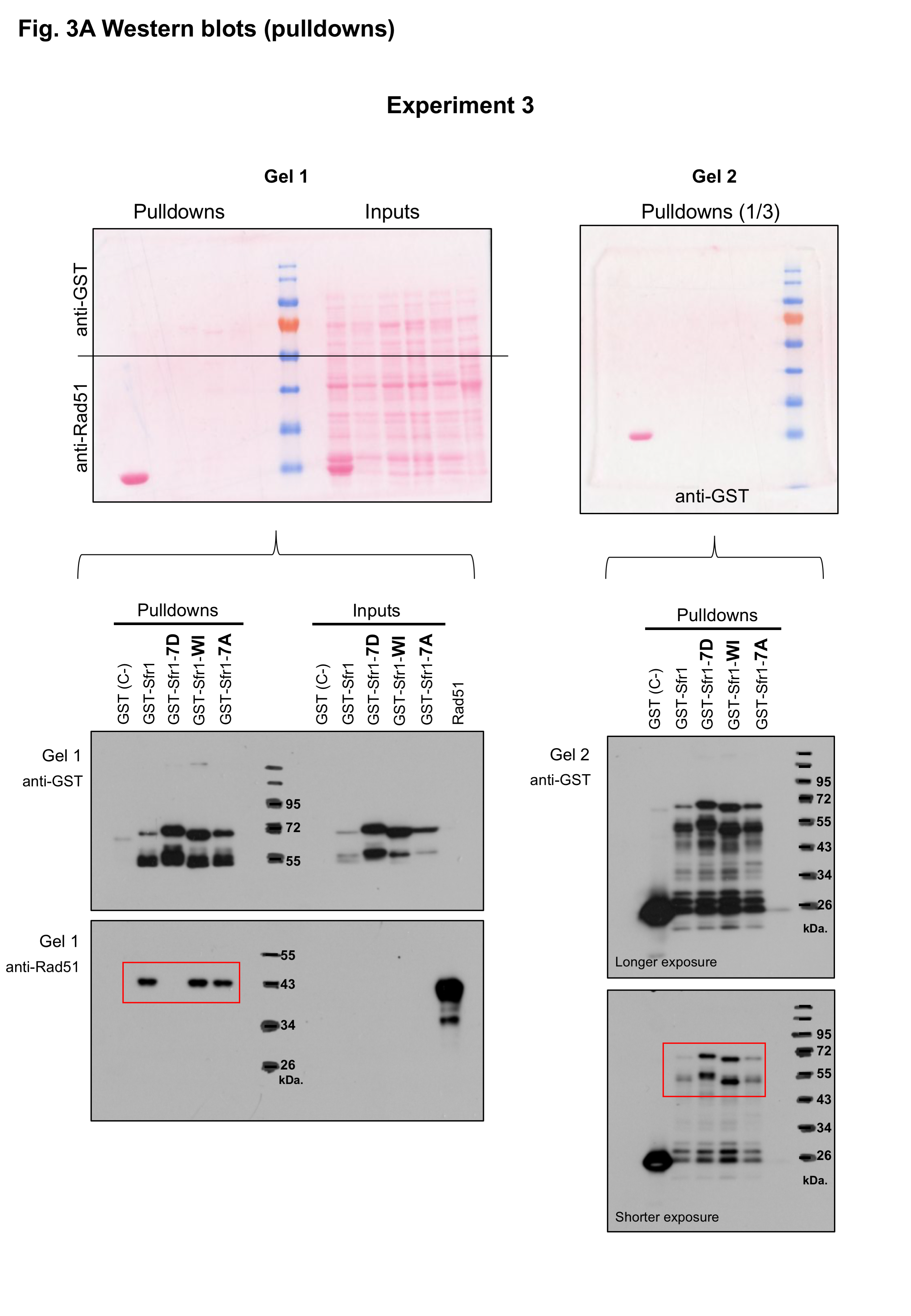

Supplement: Supplementary file 18 — Source data Fig. 3 [file 44318_2024_205_MOESM18_ESM.zip › Figure 3 Source Data/3A/3A Western blots/Experiment 3.tiff]

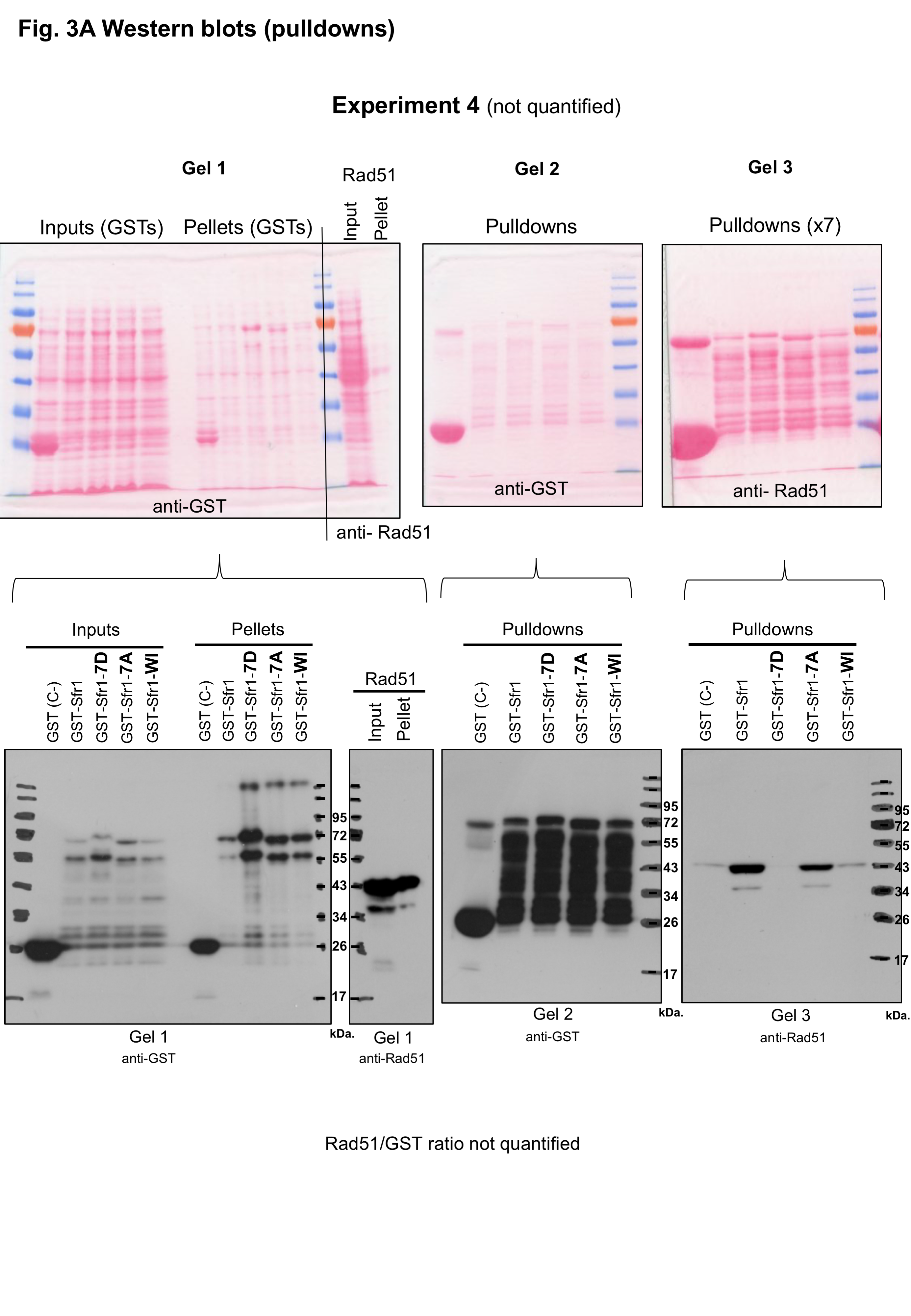

Supplement: Supplementary file 18 — Source data Fig. 3 [file 44318_2024_205_MOESM18_ESM.zip › Figure 3 Source Data/3A/3A Western blots/Experiment 4.tiff]

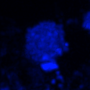

Supplement: Supplementary file 19 — Source data Fig. 4 [file 44318_2024_205_MOESM19_ESM.zip › Figure 4 Source Data/4A/4A images/control EGFP-Sfr1/C1(DNA)-MAX_1649 3.5.tif]

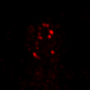

Supplement: Supplementary file 19 — Source data Fig. 4 [file 44318_2024_205_MOESM19_ESM.zip › Figure 4 Source Data/4A/4A images/control EGFP-Sfr1/C2(Rad51)-MAX_1649 3.5.tif]

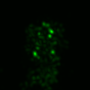

Supplement: Supplementary file 19 — Source data Fig. 4 [file 44318_2024_205_MOESM19_ESM.zip › Figure 4 Source Data/4A/4A images/control EGFP-Sfr1/C3(EGFP-Sfr1)-MAX_1649 3.5.tif]

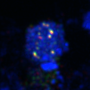

Supplement: Supplementary file 19 — Source data Fig. 4 [file 44318_2024_205_MOESM19_ESM.zip › Figure 4 Source Data/4A/4A images/control EGFP-Sfr1/MAX_1649 3.5 (RGB).tif]

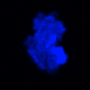

Supplement: Supplementary file 19 — Source data Fig. 4 [file 44318_2024_205_MOESM19_ESM.zip › Figure 4 Source Data/4A/4A images/EGFP-Sfr1-7A/C1(DNA)-MAX_1733 3.5h.tif]

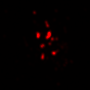

Supplement: Supplementary file 19 — Source data Fig. 4 [file 44318_2024_205_MOESM19_ESM.zip › Figure 4 Source Data/4A/4A images/EGFP-Sfr1-7A/C2(Rad51)-MAX_1733 3.5h.tif]

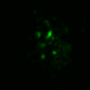

Supplement: Supplementary file 19 — Source data Fig. 4 [file 44318_2024_205_MOESM19_ESM.zip › Figure 4 Source Data/4A/4A images/EGFP-Sfr1-7A/C3(EGFP-Sfr1-7A)-MAX_1733 3.5.tif]

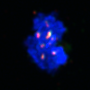

Supplement: Supplementary file 19 — Source data Fig. 4 [file 44318_2024_205_MOESM19_ESM.zip › Figure 4 Source Data/4A/4A images/EGFP-Sfr1-7A/MAX_1733 3.5h (RGB).tif]

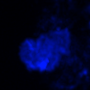

Supplement: Supplementary file 19 — Source data Fig. 4 [file 44318_2024_205_MOESM19_ESM.zip › Figure 4 Source Data/4A/4A images/EGFP-Sfr1-7D/C1(DNA)-MAX_1756 3.5h.tif]

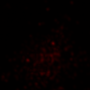

Supplement: Supplementary file 19 — Source data Fig. 4 [file 44318_2024_205_MOESM19_ESM.zip › Figure 4 Source Data/4A/4A images/EGFP-Sfr1-7D/C2(Rad51)-MAX_1756 3.5h.tif]

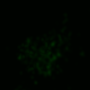

Supplement: Supplementary file 19 — Source data Fig. 4 [file 44318_2024_205_MOESM19_ESM.zip › Figure 4 Source Data/4A/4A images/EGFP-Sfr1-7D/C3(EGFP-Sfr1-7D)-MAX_1756 3.5h.tif]

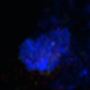

Supplement: Supplementary file 19 — Source data Fig. 4 [file 44318_2024_205_MOESM19_ESM.zip › Figure 4 Source Data/4A/4A images/EGFP-Sfr1-7D/MAX_1756 3.5h (RGB).tif]

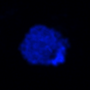

Supplement: Supplementary file 19 — Source data Fig. 4 [file 44318_2024_205_MOESM19_ESM.zip › Figure 4 Source Data/4A/4A images/EGFP-Sfr1-WI/C1(DNA)-MAX_1769 3.5h.tif]

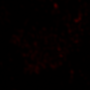

Supplement: Supplementary file 19 — Source data Fig. 4 [file 44318_2024_205_MOESM19_ESM.zip › Figure 4 Source Data/4A/4A images/EGFP-Sfr1-WI/C2(Rad51)-MAX_1769 3.5h.tif]

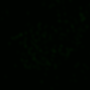

Supplement: Supplementary file 19 — Source data Fig. 4 [file 44318_2024_205_MOESM19_ESM.zip › Figure 4 Source Data/4A/4A images/EGFP-Sfr1-WI/C3(EGFP-Sfr1-WI)-MAX_1769 3.5h.tif]

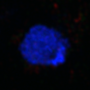

Supplement: Supplementary file 19 — Source data Fig. 4 [file 44318_2024_205_MOESM19_ESM.zip › Figure 4 Source Data/4A/4A images/EGFP-Sfr1-WI/MAX_1769 3.5h (RGB).tif]

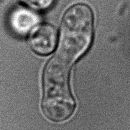

Supplement: Supplementary file 20 — Source data Fig. 5 [file 44318_2024_205_MOESM20_ESM.zip › Figure 5 Source Data/5A/5A images/control EGFP-Sfr1/EGFP-Sfr1 TRANS.tif]

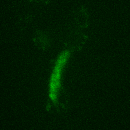

Supplement: Supplementary file 20 — Source data Fig. 5 [file 44318_2024_205_MOESM20_ESM.zip › Figure 5 Source Data/5A/5A images/control EGFP-Sfr1/MAX_20230519 EGFP-Sfr1 -100 min.tif]

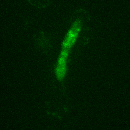

Supplement: Supplementary file 20 — Source data Fig. 5 [file 44318_2024_205_MOESM20_ESM.zip › Figure 5 Source Data/5A/5A images/control EGFP-Sfr1/MAX_20230519 EGFP-Sfr1 -70 min.tif]

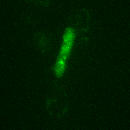

Supplement: Supplementary file 20 — Source data Fig. 5 [file 44318_2024_205_MOESM20_ESM.zip › Figure 5 Source Data/5A/5A images/control EGFP-Sfr1/MAX_20230519 EGFP-Sfr1 -80 min.tif]

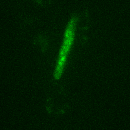

Supplement: Supplementary file 20 — Source data Fig. 5 [file 44318_2024_205_MOESM20_ESM.zip › Figure 5 Source Data/5A/5A images/control EGFP-Sfr1/MAX_20230519 EGFP-Sfr1 -90 min.tif]

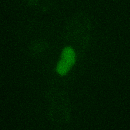

Supplement: Supplementary file 20 — Source data Fig. 5 [file 44318_2024_205_MOESM20_ESM.zip › Figure 5 Source Data/5A/5A images/control EGFP-Sfr1/MAX_20230519 EGFP-Sfr1 0 min.tif]

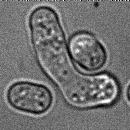

Supplement: Supplementary file 20 — Source data Fig. 5 [file 44318_2024_205_MOESM20_ESM.zip › Figure 5 Source Data/5A/5A images/EGFP-Sfr1 in delta rec12/EGFP-Sfr1 delta rec12 TRANS.tif]

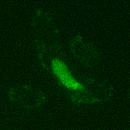

Supplement: Supplementary file 20 — Source data Fig. 5 [file 44318_2024_205_MOESM20_ESM.zip › Figure 5 Source Data/5A/5A images/EGFP-Sfr1 in delta rec12/MAX_20221103 EGFP-Sfr1 delta rec12 -100 min.tif]

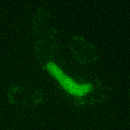

Supplement: Supplementary file 20 — Source data Fig. 5 [file 44318_2024_205_MOESM20_ESM.zip › Figure 5 Source Data/5A/5A images/EGFP-Sfr1 in delta rec12/MAX_20221103 EGFP-Sfr1 delta rec12 -70 min.tif]

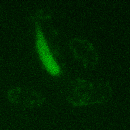

Supplement: Supplementary file 20 — Source data Fig. 5 [file 44318_2024_205_MOESM20_ESM.zip › Figure 5 Source Data/5A/5A images/EGFP-Sfr1 in delta rec12/MAX_20221103 EGFP-Sfr1 delta rec12 -80 min.tif]

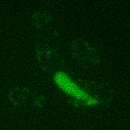

Supplement: Supplementary file 20 — Source data Fig. 5 [file 44318_2024_205_MOESM20_ESM.zip › Figure 5 Source Data/5A/5A images/EGFP-Sfr1 in delta rec12/MAX_20221103 EGFP-Sfr1 delta rec12 -90 min.tif]

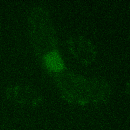

Supplement: Supplementary file 20 — Source data Fig. 5 [file 44318_2024_205_MOESM20_ESM.zip › Figure 5 Source Data/5A/5A images/EGFP-Sfr1 in delta rec12/MAX_20221103 EGFP-Sfr1 delta rec12 0 min.tif]

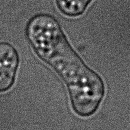

Supplement: Supplementary file 20 — Source data Fig. 5 [file 44318_2024_205_MOESM20_ESM.zip › Figure 5 Source Data/5A/5A images/EGFP-Sfr1-7A/EGFP-Sfr1-7A TRANS.tif]

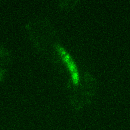

Supplement: Supplementary file 20 — Source data Fig. 5 [file 44318_2024_205_MOESM20_ESM.zip › Figure 5 Source Data/5A/5A images/EGFP-Sfr1-7A/MAX_20221103 EGFP-Sfr1-7A -100 min.tif]

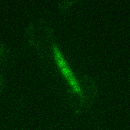

Supplement: Supplementary file 20 — Source data Fig. 5 [file 44318_2024_205_MOESM20_ESM.zip › Figure 5 Source Data/5A/5A images/EGFP-Sfr1-7A/MAX_20221103 EGFP-Sfr1-7A -70 min.tif]

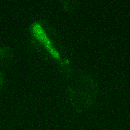

Supplement: Supplementary file 20 — Source data Fig. 5 [file 44318_2024_205_MOESM20_ESM.zip › Figure 5 Source Data/5A/5A images/EGFP-Sfr1-7A/MAX_20221103 EGFP-Sfr1-7A -80 min.tif]

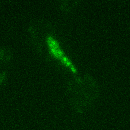

Supplement: Supplementary file 20 — Source data Fig. 5 [file 44318_2024_205_MOESM20_ESM.zip › Figure 5 Source Data/5A/5A images/EGFP-Sfr1-7A/MAX_20221103 EGFP-Sfr1-7A -90 min.tif]

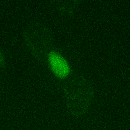

Supplement: Supplementary file 20 — Source data Fig. 5 [file 44318_2024_205_MOESM20_ESM.zip › Figure 5 Source Data/5A/5A images/EGFP-Sfr1-7A/MAX_20221103 EGFP-Sfr1-7A 0 min.tif]

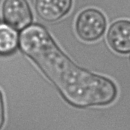

Supplement: Supplementary file 20 — Source data Fig. 5 [file 44318_2024_205_MOESM20_ESM.zip › Figure 5 Source Data/5A/5A images/EGFP-Sfr1-7D/EGFP-Sfr1-7D TRANS.tif]

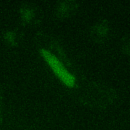

Supplement: Supplementary file 20 — Source data Fig. 5 [file 44318_2024_205_MOESM20_ESM.zip › Figure 5 Source Data/5A/5A images/EGFP-Sfr1-7D/MAX_20230420 EGFP-Sfr1-7D -100 min.tif]

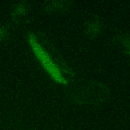

Supplement: Supplementary file 20 — Source data Fig. 5 [file 44318_2024_205_MOESM20_ESM.zip › Figure 5 Source Data/5A/5A images/EGFP-Sfr1-7D/MAX_20230420 EGFP-Sfr1-7D -70 min.tif]

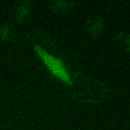

Supplement: Supplementary file 20 — Source data Fig. 5 [file 44318_2024_205_MOESM20_ESM.zip › Figure 5 Source Data/5A/5A images/EGFP-Sfr1-7D/MAX_20230420 EGFP-Sfr1-7D -80 min.tif]

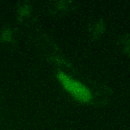

Supplement: Supplementary file 20 — Source data Fig. 5 [file 44318_2024_205_MOESM20_ESM.zip › Figure 5 Source Data/5A/5A images/EGFP-Sfr1-7D/MAX_20230420 EGFP-Sfr1-7D -90 min.tif]

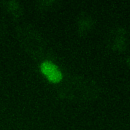

Supplement: Supplementary file 20 — Source data Fig. 5 [file 44318_2024_205_MOESM20_ESM.zip › Figure 5 Source Data/5A/5A images/EGFP-Sfr1-7D/MAX_20230420 EGFP-Sfr1-7D 0 min.tif]

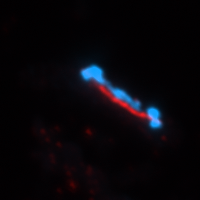

Supplement: Supplementary file 22 — Source data Fig. 7 [file 44318_2024_205_MOESM22_ESM.zip › Figure 7 Source Data/7A/7A images/Abnormal segregations/Anaphase I/zygote 1/Anaphase I-1 (RGB).tif]

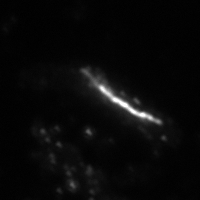

Supplement: Supplementary file 22 — Source data Fig. 7 [file 44318_2024_205_MOESM22_ESM.zip › Figure 7 Source Data/7A/7A images/Abnormal segregations/Anaphase I/zygote 1/C1(tubulin)-Anaphase I-1.tif]

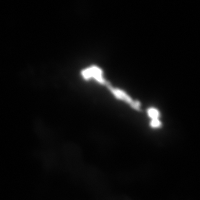

Supplement: Supplementary file 22 — Source data Fig. 7 [file 44318_2024_205_MOESM22_ESM.zip › Figure 7 Source Data/7A/7A images/Abnormal segregations/Anaphase I/zygote 1/C2(DNA)-Anaphase I-1.tif]

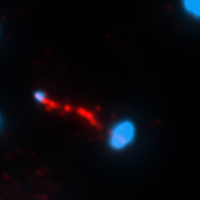

Supplement: Supplementary file 22 — Source data Fig. 7 [file 44318_2024_205_MOESM22_ESM.zip › Figure 7 Source Data/7A/7A images/Abnormal segregations/Anaphase I/zygote 2/Anaphase I-2 (RGB).tif]

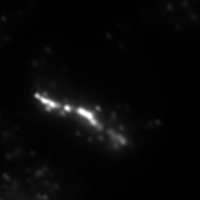

Supplement: Supplementary file 22 — Source data Fig. 7 [file 44318_2024_205_MOESM22_ESM.zip › Figure 7 Source Data/7A/7A images/Abnormal segregations/Anaphase I/zygote 2/C1(tubulin)-Anaphase I-2.tif]

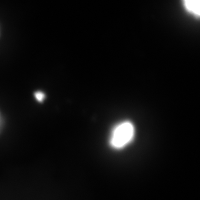

Supplement: Supplementary file 22 — Source data Fig. 7 [file 44318_2024_205_MOESM22_ESM.zip › Figure 7 Source Data/7A/7A images/Abnormal segregations/Anaphase I/zygote 2/C2(DNA)-Anaphase I-2.tif]

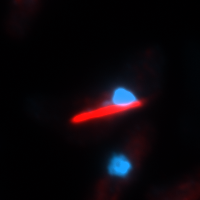

Supplement: Supplementary file 22 — Source data Fig. 7 [file 44318_2024_205_MOESM22_ESM.zip › Figure 7 Source Data/7A/7A images/Abnormal segregations/Anaphase I/zygote 3/Anaphase I-3 (RGB).tif]

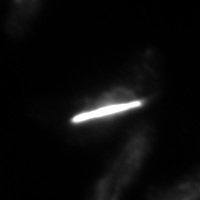

Supplement: Supplementary file 22 — Source data Fig. 7 [file 44318_2024_205_MOESM22_ESM.zip › Figure 7 Source Data/7A/7A images/Abnormal segregations/Anaphase I/zygote 3/C1(tubulin)-Anaphase I-3.tif]

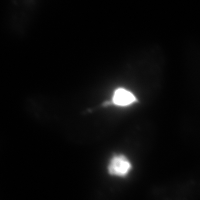

Supplement: Supplementary file 22 — Source data Fig. 7 [file 44318_2024_205_MOESM22_ESM.zip › Figure 7 Source Data/7A/7A images/Abnormal segregations/Anaphase I/zygote 3/C2(DNA)-Anaphase I-3.tif]

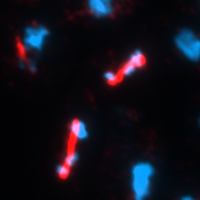

Supplement: Supplementary file 22 — Source data Fig. 7 [file 44318_2024_205_MOESM22_ESM.zip › Figure 7 Source Data/7A/7A images/Abnormal segregations/Anaphase II/zygote 1/Anaphase II-1 (RGB).tif]

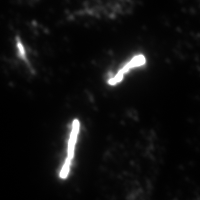

Supplement: Supplementary file 22 — Source data Fig. 7 [file 44318_2024_205_MOESM22_ESM.zip › Figure 7 Source Data/7A/7A images/Abnormal segregations/Anaphase II/zygote 1/C1(tubulin)-Anaphase II-1.tif]

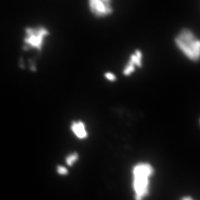

Supplement: Supplementary file 22 — Source data Fig. 7 [file 44318_2024_205_MOESM22_ESM.zip › Figure 7 Source Data/7A/7A images/Abnormal segregations/Anaphase II/zygote 1/C2(DNA) Anaphase II-1.tif]

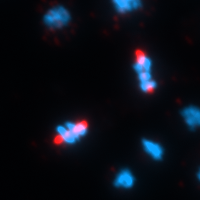

Supplement: Supplementary file 22 — Source data Fig. 7 [file 44318_2024_205_MOESM22_ESM.zip › Figure 7 Source Data/7A/7A images/Abnormal segregations/Anaphase II/zygote 2/Anaphase II-2 (RGB).tif]

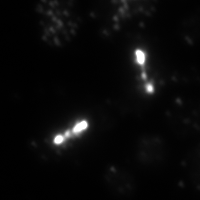

Supplement: Supplementary file 22 — Source data Fig. 7 [file 44318_2024_205_MOESM22_ESM.zip › Figure 7 Source Data/7A/7A images/Abnormal segregations/Anaphase II/zygote 2/C1(tubulin)-Anaphase II-2.tif]

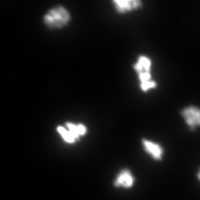

Supplement: Supplementary file 22 — Source data Fig. 7 [file 44318_2024_205_MOESM22_ESM.zip › Figure 7 Source Data/7A/7A images/Abnormal segregations/Anaphase II/zygote 2/C2(DNA)-Anaphase II-2.tif]

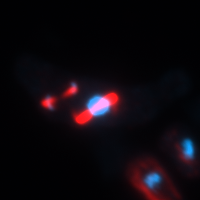

Supplement: Supplementary file 22 — Source data Fig. 7 [file 44318_2024_205_MOESM22_ESM.zip › Figure 7 Source Data/7A/7A images/Abnormal segregations/Anaphase II/zygote 3/Anaphase II-3 (RGB).tif]

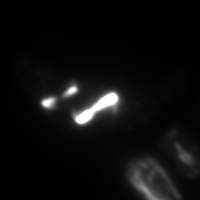

Supplement: Supplementary file 22 — Source data Fig. 7 [file 44318_2024_205_MOESM22_ESM.zip › Figure 7 Source Data/7A/7A images/Abnormal segregations/Anaphase II/zygote 3/C1(tubulin)-Anaphase II-3.tif]

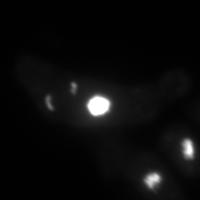

Supplement: Supplementary file 22 — Source data Fig. 7 [file 44318_2024_205_MOESM22_ESM.zip › Figure 7 Source Data/7A/7A images/Abnormal segregations/Anaphase II/zygote 3/C2(DNA)-Anaphase II-3.tif]

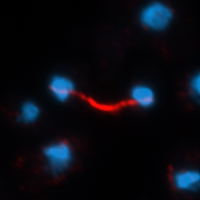

Supplement: Supplementary file 22 — Source data Fig. 7 [file 44318_2024_205_MOESM22_ESM.zip › Figure 7 Source Data/7A/7A images/Proper segregations/Anaphase I/Anaphase I (RGB).tif]

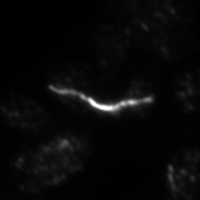

Supplement: Supplementary file 22 — Source data Fig. 7 [file 44318_2024_205_MOESM22_ESM.zip › Figure 7 Source Data/7A/7A images/Proper segregations/Anaphase I/C1(tubulin)-Anaphase I.tif]

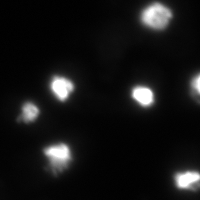

Supplement: Supplementary file 22 — Source data Fig. 7 [file 44318_2024_205_MOESM22_ESM.zip › Figure 7 Source Data/7A/7A images/Proper segregations/Anaphase I/C2(DNA)-Anaphase I.tif]

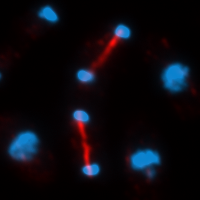

Supplement: Supplementary file 22 — Source data Fig. 7 [file 44318_2024_205_MOESM22_ESM.zip › Figure 7 Source Data/7A/7A images/Proper segregations/Anaphase II/Anaphase II (RGB).tif]

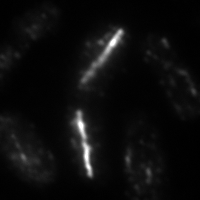

Supplement: Supplementary file 22 — Source data Fig. 7 [file 44318_2024_205_MOESM22_ESM.zip › Figure 7 Source Data/7A/7A images/Proper segregations/Anaphase II/C1(tubulin)-Anaphase II.tif]

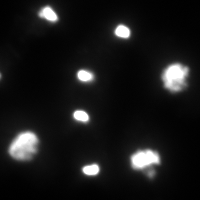

Supplement: Supplementary file 22 — Source data Fig. 7 [file 44318_2024_205_MOESM22_ESM.zip › Figure 7 Source Data/7A/7A images/Proper segregations/Anaphase II/C2(DNA)-Anaphase II.tif]

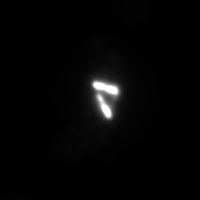

Supplement: Supplementary file 22 — Source data Fig. 7 [file 44318_2024_205_MOESM22_ESM.zip › Figure 7 Source Data/7B/7B images/C1(tubulin)-single DNA mass and two spindles.tif]

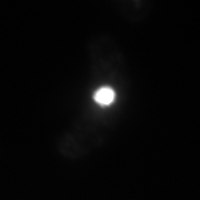

Supplement: Supplementary file 22 — Source data Fig. 7 [file 44318_2024_205_MOESM22_ESM.zip › Figure 7 Source Data/7B/7B images/C2(DNA)-single DNA mass and two spindles.tif]

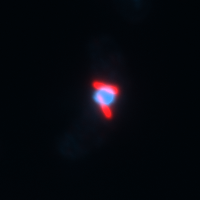

Supplement: Supplementary file 22 — Source data Fig. 7 [file 44318_2024_205_MOESM22_ESM.zip › Figure 7 Source Data/7B/7B images/single DNA mass and two spindles (RGB).tif]

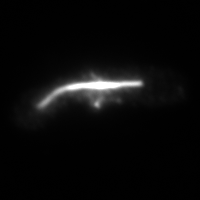

Supplement: Supplementary file 22 — Source data Fig. 7 [file 44318_2024_205_MOESM22_ESM.zip › Figure 7 Source Data/7C/7C images/C1(tubulin)-Rad51 persistance.tif]

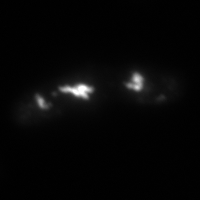

Supplement: Supplementary file 22 — Source data Fig. 7 [file 44318_2024_205_MOESM22_ESM.zip › Figure 7 Source Data/7C/7C images/C2(DNA)-Rad51 persistance.tif]

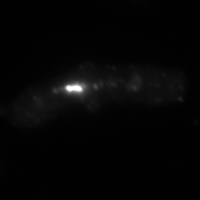

Supplement: Supplementary file 22 — Source data Fig. 7 [file 44318_2024_205_MOESM22_ESM.zip › Figure 7 Source Data/7C/7C images/C3(Rad51)-Rad51 persistance.tif]

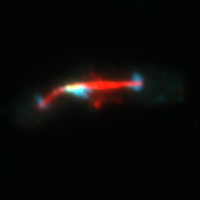

Supplement: Supplementary file 22 — Source data Fig. 7 [file 44318_2024_205_MOESM22_ESM.zip › Figure 7 Source Data/7C/7C images/Rad51 persistance (RGB).tif]
