# Supplementary material for: CDK phosphorylation of Sfr1 downregulates Rad51 function in late-meiotic homolog invasions
Source: EMBO J. 2024 Aug 22;43(19):4356–83. doi: 10.1038/s44318-024-00205-2 (PMC11445502; doi:10.1038/s44318-024-00205-2)
Supplement: Supplementary file 23 — Expanded View Figures [file 44318_2024_205_MOESM23_ESM.pdf]

Expanded View Figures

A

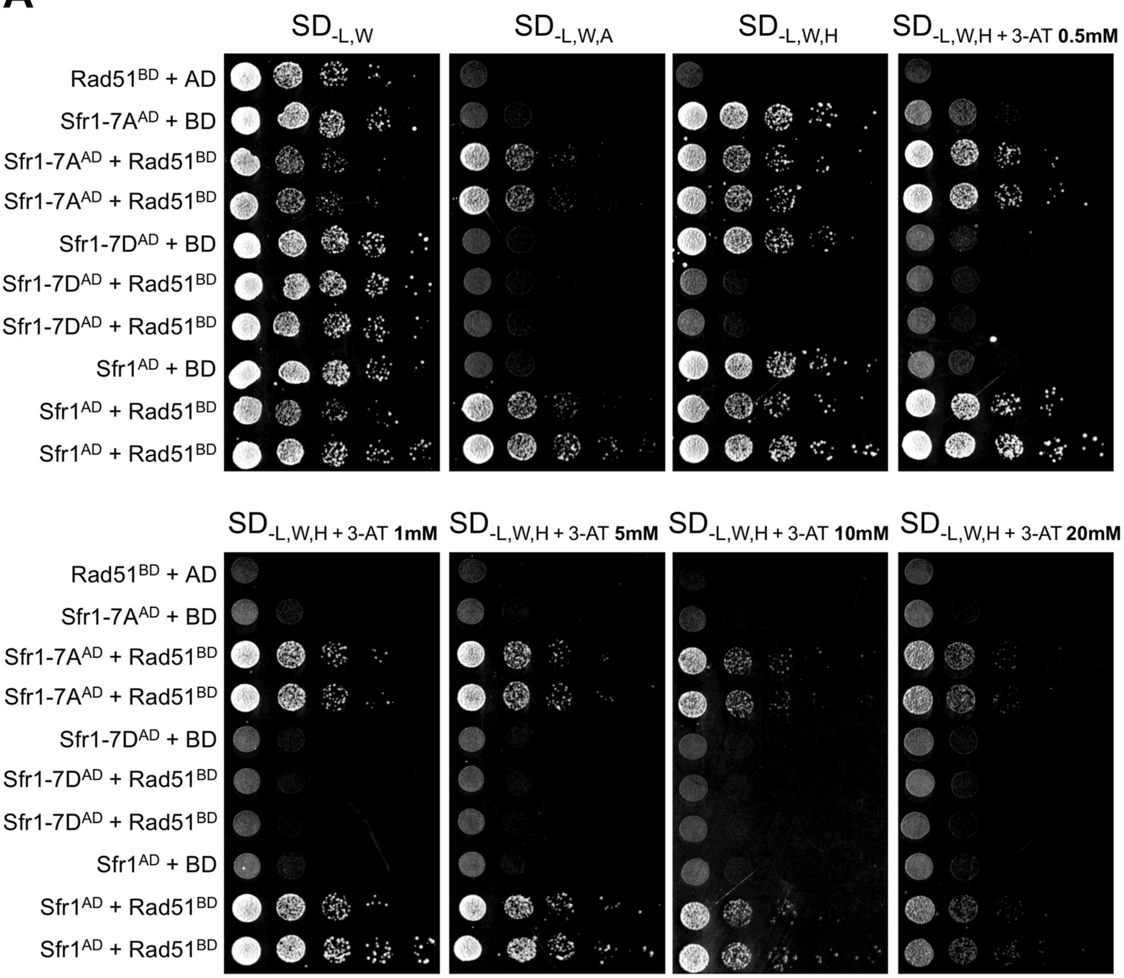

B

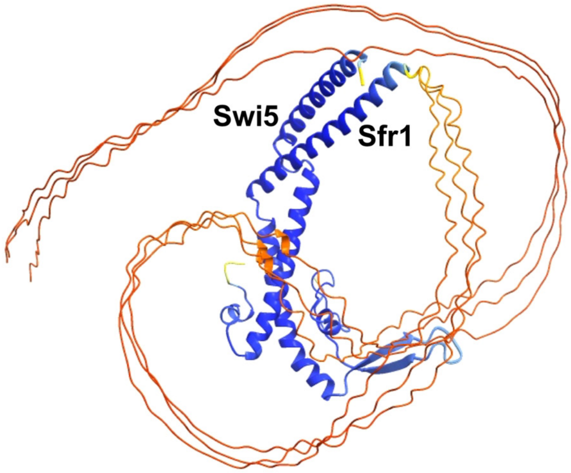

© The Author(s)

Figure EV1. Sfr1-7A but not Sfr1-7D interacts with Rad51 in Yeast Two-Hybrid assays.

(**A**) Strains expressing Sfr1, Sfr1-7A, and Sfr1-7D fused to the GAL4 transcription-activation domain and Rad51 fused to the GAL4 DNA-binding domain were grown on SD plates lacking tryptophan and leucine (SD-L,W), grown in SD-L,W liquid O/N at 30 °C and then spotted at 10-fold serial dilutions on SD plates lacking leucine and tryptophan (SD-L,W) and SD plates lacking leucine, tryptophan and adenine (SD-L,W,A) or SD plates lacking leucine, tryptophan and histidine (SD-L,W,H) (with or without different concentrations of 3-amino-1,2,4-triazol, 3-AT). The known interaction between Sfr1 and Rad51 was used as a positive control. Growth on plates without histidine or without adenine indicates interaction between the fusion proteins. 3-AT is a competitive inhibitor of *HIS3* gene product which inhibits non-specific interactions and helps to discriminate differences in the interaction efficiency. Two independent transformants are shown for each interaction. Related to Fig. 3A. (**B**) Swi5–Sfr1 complexes with wild-type, Sfr1-7A and Sfr1-7D proteins modeled by ColabFold are shown (color bfactor palette) (https://colab.research.google.com/github/sokrypton/ColabFold/blob/main/AlphaFold2.ipynb). The large disordered N-terminal part of the proteins are shown in orange.

 © The Author(s)

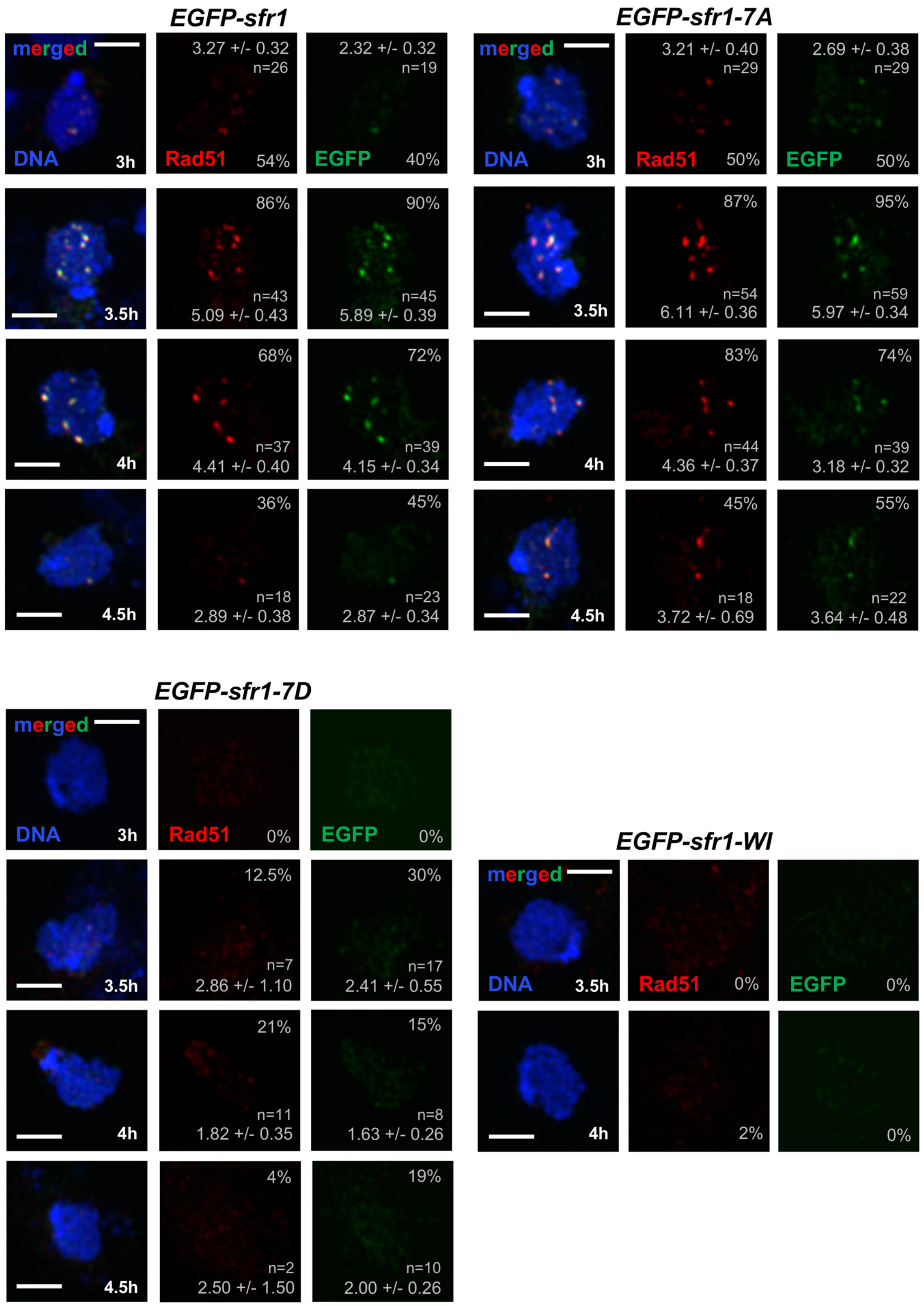
EGFP-sfr1
merged
DNA
3h
Rad51
3.27 +/- 0.32
n=26
54%
EGFP
2.32 +/- 0.32
n=19
40%
3.5h
86%
n=43
5.09 +/- 0.43
90%
n=45
5.89 +/- 0.39
4h
68%
n=37
4.41 +/- 0.40
72%
n=39
4.15 +/- 0.34
4.5h
36%
n=18
2.89 +/- 0.38
45%
n=23
2.87 +/- 0.34
EGFP-sfr1-7A
merged
DNA
3h
Rad51
3.21 +/- 0.40
n=29
50%
EGFP
2.69 +/- 0.38
n=29
50%
3.5h
87%
n=54
6.11 +/- 0.36
95%
n=59
5.97 +/- 0.34
4h
83%
n=44
4.36 +/- 0.37
74%
n=39
3.18 +/- 0.32
4.5h
45%
n=18
3.72 +/- 0.69
55%
n=22
3.64 +/- 0.48
EGFP-sfr1-7D
merged
DNA
3h
Rad51
0%
EGFP
0%
3.5h
12.5%
n=7
2.86 +/- 1.10
30%
n=17
2.41 +/- 0.55
4h
21%
n=11
1.82 +/- 0.35
15%
n=8
1.63 +/- 0.26
4.5h
4%
n=2
2.50 +/- 1.50
19%
n=10
2.00 +/- 0.26
EGFP-sfr1-WI
merged
DNA
3.5h
Rad51
0%
EGFP
0%
4h
2%
0%

© The Author(s)

Figure EV2. Localization of mutant Sfr1 proteins and impact on Rad51 chromosome loading.

pat1-114 EGFP-sfr1 (CMC1649), *EGFP-sfr1-7A* (CMC1733), *EGFP-sfr1-7D* (CMC1756) and *EGFP-sfr1-WI* (CMC1769) diploid cells were induced to enter meiosis and collected at different time points during prophase for nuclear spread preparation. Spreads were stained with anti-GFP antibodies for the visualization of EGFP-Sfr1 proteins (in green) and with anti-Rad51 antibodies (in red). DAPI staining to visualize DNA is shown in blue. Representative images of nuclei at each time point after meiotic induction are shown (maximum Z projections). Notice that the images at 3.5 h are also presented as an introductory summary of the experiment in Fig. 4A. Scale bars correspond to 2 µm. The percentages of nuclei showing foci for the corresponding proteins are indicated. The mean of the number of foci per nucleus +/− SEM (in n analyzed nuclei) is also indicated. Related to Fig. 4.

 © The Author(s)

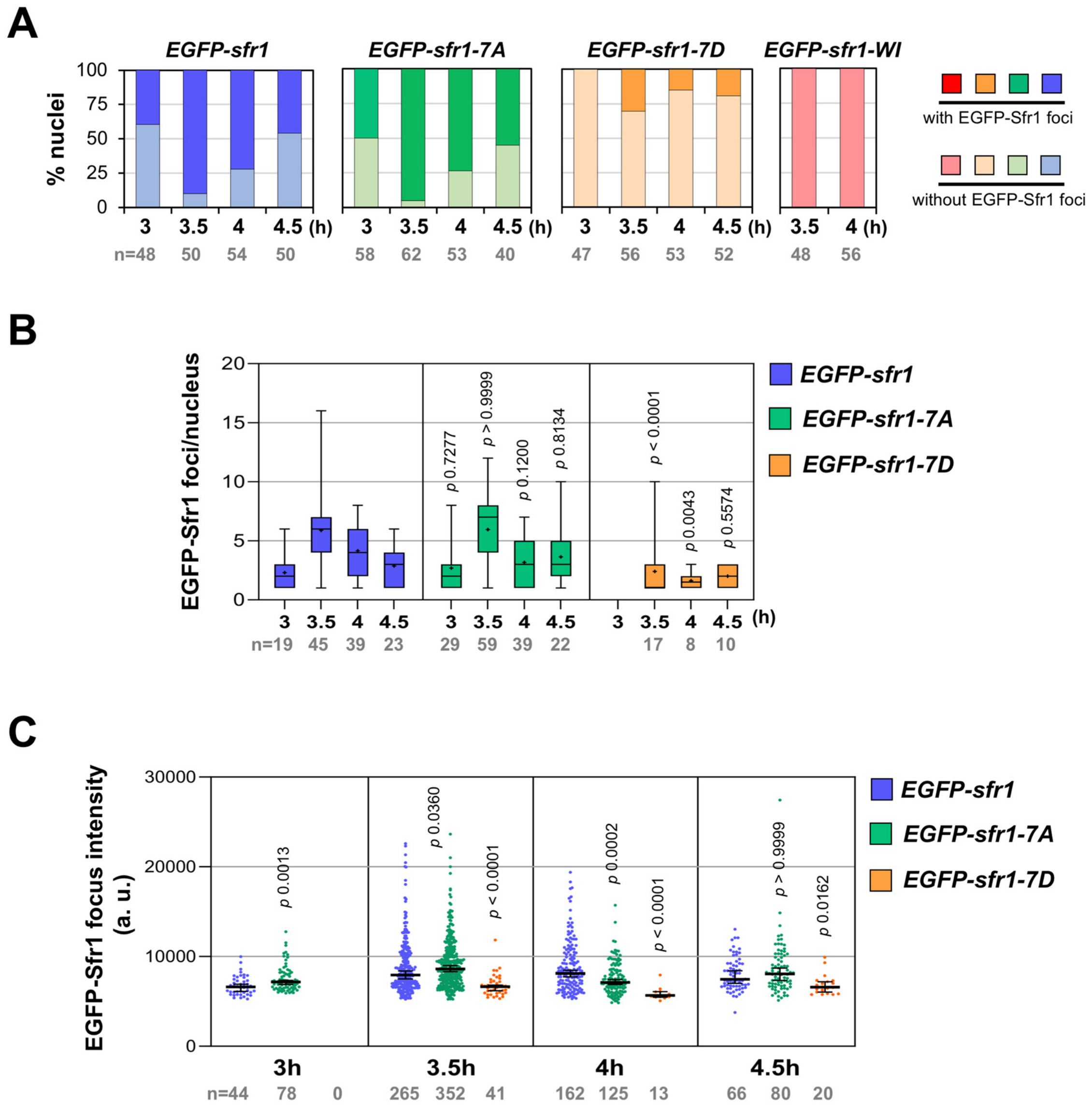

Figure EV3. Impact of Sfr1 phosphorylation on its chromatin loading.

pat1-114 EGFP-sfr1 (CMC1649), *EGFP-sfr1-7A* (CMC1733), *EGFP-sfr1-7D* (CMC1756) and *EGFP-sfr1-WI* (CMC1769) diploid cells were induced to enter meiosis and collected at different time points during prophase for nuclear spread preparation. Spreads were stained with anti-GFP antibodies for the visualization of EGFP-Sfr1 proteins. (**A**) Dynamics of EGFP-Sfr1 (or mutant versions) signals during prophase. The percentage of nuclei with (dark colors) and without (light colors) EGFP-Sfr1 foci is represented. (**B**) Quantification of the number of EGFP-Sfr1 (or mutant versions) foci per nucleus in the nuclei with signal. Data are represented by box-and-whisker plots where boxes extend from the 25th to 75th percentiles, and bars within the boxes represent the medians and black crosses the means; the whiskers represent the minimum and the maximal range. (**C**) Representation of the intensity of individual EGFP-Sfr1 (or mutant versions) foci. The median $+/-$ 95% confidence interval is indicated. For all the graphs in the figure, the number of analyzed nuclei or foci is indicated (*n*). Comparisons were done with the *EGFP-sfr1* control experiment, and *P* values were calculated based on Mann–Whitney test to compare results at 3 h (2 groups) and Kruskal–Wallis test (one-way nonparametric ANOVA) with Dunn's correction at the rest of the time points. Related to Fig. 4.

© The Author(s)

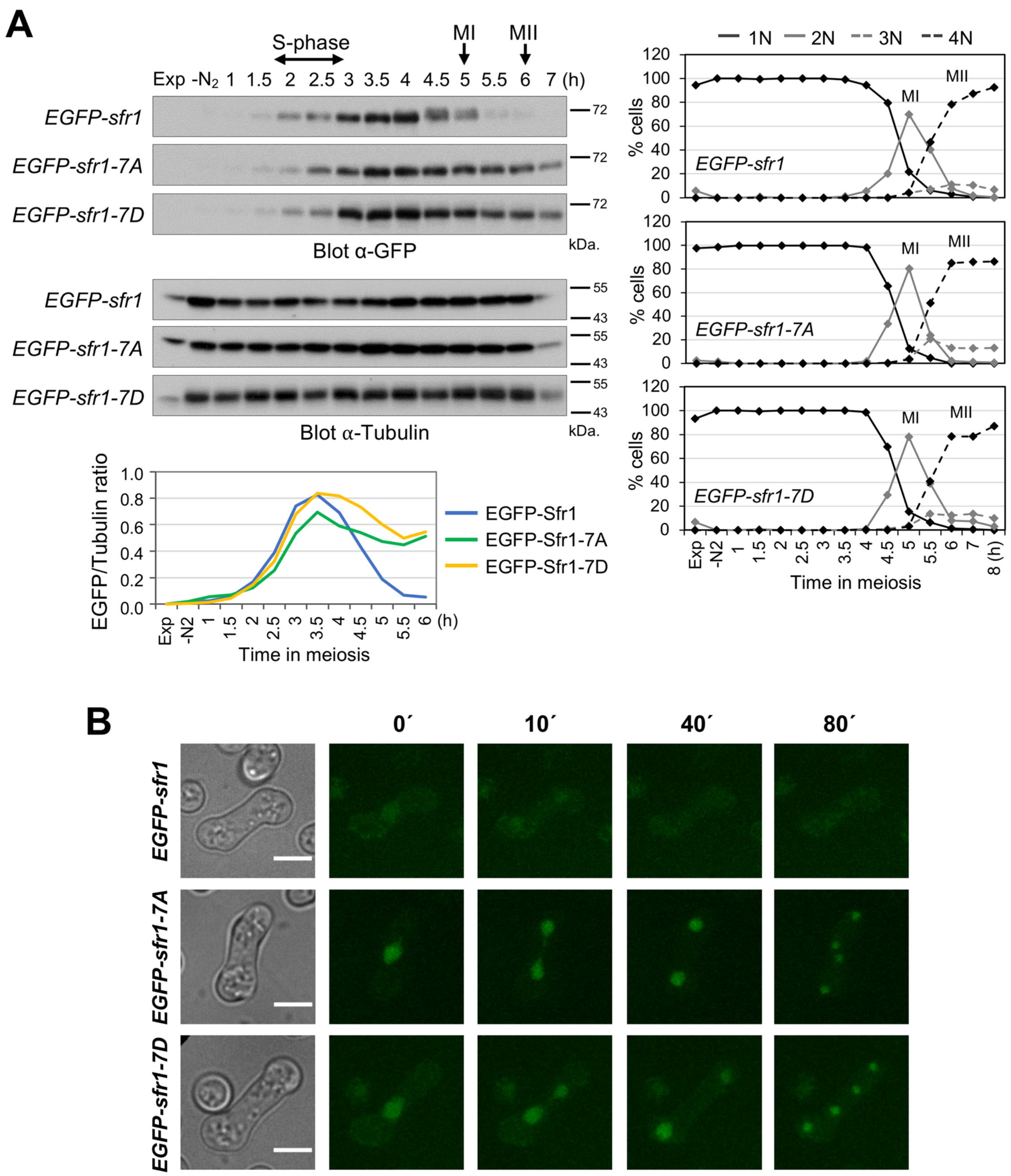

Figure EV4. Expression of EGFP-Sfr1 and phospho-mutant proteins.

(**A**) *pat1-114 EGFP-sfr1* (CMC1649), *EGFP-sfr1-7A* (CMC1733) and *EGFP-sfr1-7D* (CMC1756) diploid cells were induced to enter meiosis and collected at the indicated time points. Left, western blot detection of EGFP-Sfr1, EGFP-Sfr1-7A and EGFP-Sfr1-7D proteins (upper blots); tubulin detection was used as loading control (lower blots). Quantification of EGFP levels is shown at the bottom (EGFP/tubulin ratio, mean of two independent kinetics). Right, meiotic progression measured as the number of nuclei per cell; timing of meiosis I (MI) and meiosis II (MII) is indicated. (**B**) Same time lapse experiments as in Fig. 5 showing EGFP-Sfr1-7A and EGFP-Sfr1-7D expression after prophase. In this representation, time point 0 was set as the frame just prior to the first meiotic division (meiosis I) detected by the EGFP signal in the segregating nucleus. Scale bars correspond to 5 μm. Related to Fig. 5.

 © The Author(s)
